# Evolution of a Large, Conserved, and Syntenic Gene Family in Insects

**DOI:** 10.1534/g3.111.001412

**Published:** 2012-02-01

**Authors:** Neethu Shah, Douglas R. Dorer, Etsuko N. Moriyama, Alan C. Christensen

**Affiliations:** * Department of Computer Science and Engineering, University of Nebraska–Lincoln, Lincoln, Nebraska 68588-0115; † Division of Mathematics & Sciences, Martin Methodist College, Pulaski, Tennessee 38478-2716; ‡ Center for Plant Science Innovation; § School of Biological Sciences, University of Nebraska–Lincoln, Lincoln, Nebraska 68588-0666

**Keywords:** gene duplication, insect, gene family, synteny, *Osiris*, triplo-lethal

## Abstract

The *Osiris* gene family, first described in *Drosophila melanogaster*, is clustered in the genomes of all *Drosophila* species sequenced to date. In *D. melanogaster*, it explains the enigmatic phenomenon of the triplo-lethal and haploinsufficient locus *Tpl*. The synteny of *Osiris* genes in flies is well conserved, and it is one of the largest syntenic blocks in the *Drosophila* group. By examining the genome sequences of other insects in a wide range of taxonomic orders, we show here that the gene family is well-conserved and syntenic not only in the diptera but across the holometabolous and hemimetabolous insects. *Osiris* gene homologs have also been found in the expressed sequence tag sequences of various other insects but are absent from all groups that are not insects, including crustacea and arachnids. It is clear that the gene family evolved by gene duplication and neofunctionalization very soon after the divergence of the insects from other arthropods but before the divergence of the insects from one another and that the sequences and synteny have been maintained by selection ever since.

Gene families are commonly found in genomes and are thought to evolve by gene duplication and neofunctionalization. The *Osiris* gene family is a large conserved family first described in *Drosophila melanogaster* (Dorer *et al.* 2003). Although the genes are still of unknown function, they are the molecular basis of the unique *Triplo-lethal* locus in *D. melanogaster*, first described in 1972 (Lindsley *et al.* 1972). The proteins have a secretion signal peptide and four domains that identify them as *Osiris* family members, one of those being a putative transmembrane domain. Twenty-three *Osiris* genes were originally found in the *D. melanogaster* genome, with 20 of them located on chromosome 3R (83E) in a cluster within a 168-kb region, which is both triplo-lethal and haplo-lethal. The *Osiris* gene family was also found in the mosquito *Anopheles gambiae*, maintaining the synteny except for a chromosomal rearrangement that split the cluster (Dorer *et al.* 2003). Subsequent work revealed that the synteny was strongly conserved among 12 diverse *Drosophila* species (Bhutkar *et al.* 2008). In this work, we report the existence of the *Osiris* gene family, which now includes 24 orthologous gene groups, in a diverse group of insects and report on the evolution of the genes and the conservation of the synteny during a very long evolutionary time frame. The interrupted synteny seen in *Anopheles gambiae* is the exception rather than the rule, and we show that the *Osiris* gene cluster is a well-conserved, insect-specific, and remarkably syntenic gene family.

## Materials and Methods

### Osiris protein sequences used

The 24 *D. melanogaster* Osiris protein sequences were downloaded from Flybase (http://flybase.org/). Their annotation symbols (CG numbers) are listed in supporting information, Table S1. These protein sequences were used as the queries for searching *Osiris* genes in various organisms.

### Insect and other arthropod genomes used

Insect and other arthropod genomes were downloaded from various sources as listed in Table S2. *Daphnia pulex* (a water flea, Subphylum Crustacea) and *Ixodes scapularis* (the deer tick, Class Arachnida) are the two noninsect Arthropoda in which the sequences of complete genomes are available. For insects, we examined in total 23 complete genomes, including 12 species of *Drosophila*, three species of mosquito (*Anopheles gambiae*, *Culex quinquefasciatus*, and *Aedes aegypti*), two hymenoptera (*Apis mellifera* and *Camponotus floridanus*), one coleopteran (*Tribolium castaneum*), one lepidopteran (*Bombyx mori*), one phthirapteran (*Pediculus humanus*), and one hemipteran (*Acyrthosiphon pisum*).

### BLAST similarity search against NCBI databases

Each of the 24 *D. melanogaster* Osiris protein sequences was used as the query. The blastp protein similarity search (Altschul *et al.* 1997; Camacho *et al.* 2009) was performed against the nonredundant (NR) protein database at the National Center for Biotechnology and Information (NCBI) Web server (http://www.ncbi.nlm.nih.gov/BLAST/). The tblastn translated protein similarity search was also performed against NCBI’s Expressed Sequence Tag (EST) database. For both searches, the BLOSUM45 scoring matrix was used, and the E-value threshold was set at 0.01. All other options were set to the default.

### *Osiris* gene mining from complete genomes using BLAST similarity search

Using each of the 24 *D. melanogaster* Osiris protein sequences as the query, we performed blastp similarity searches against the 23 complete genomes from insects as well as *Daphnia* and *Ixodes*. The BLAST+ package (version 2.2.24+) installed on our local Linux server was used to prepare the complete set of proteins from each genome and run the blastp program. An E-value threshold of 1 was used, and the effective length of the database was set as 7,500,000 residues (on the basis of the average cumulative number of amino acid residues from all the genomes) for all genomic searches. All other options were set as the default. When gene structures given in the genome project were suspected to be incorrect or incomplete (*e.g.*, missing 5′ or 3′ exon), we used tblastn, GeneWise version 2-2-0 (Birney *et al.* 2004), and Augustus version 2.5.5 (http://bioinf.uni-greifswald.de/augustus/) (Stanke *et al.* 2008) for gene structure prediction. When gene structures were not available in the genome project, we also performed our own prediction using the same strategy. Gene structures different from the ones given in the genome projects are clarified in Table S3.

### Profile hidden Markov models (HMMs)

To perform a thorough search of *Osiris* genes, we built a profile HMM based on the alignment of all 24 Osiris proteins obtained from the aforementioned blast searches. HMMER version 3.0 (http://hmmer.janelia.org/) was used to build profile HMMs for all the 24 Osiris proteins and to perform searches using these profile HMMs against the entire protein set from each genome. The options used were hmmbuild and hmmsearch, with an E-value threshold of 0.01 and a database size of 20,000 (average of all the sequences from all the genomes). In addition to the 23 genomes, two Annelida genomes, *Capitella teleta* and *Helobdella robusta* (obtained from the Joint Genome Institute http://genome.jgi-psf.org/), were searched.

### Multiple alignments of Osiris protein sequences

Multiple alignments of Osiris protein sequences were generated using MAFFT v6.847b (Katoh and Toh 2008) with the L-INS-i algorithm. Alignments were generated individually for each Osiris group, including all Osiris sequences at once, and using the profile alignment option. The alignment of the 24 *D. melanogaster* Osiris proteins is included in Figure S1.

### Phylogenetic tree reconstruction

Phylogenetic trees were reconstructed by FastTree 2 (version 2.1.3), which can infer approximately maximum likelihood phylogenies for large data sets (Price *et al.* 2010). The default options were used except for “-gamma.” This uses the JTT+CAT (20 fixed-rate categories) model for amino acid substitutions for tree optimization and the discrete gamma model with 20 rate-categories to tree rescaling. Bootstrap analysis with 1000 pseudoreplicates was done using seqboot (Phylip version 3.68) (Felsenstein 2010) and CompareToBootstrap.pl (http://www.microbesonline.org/fasttree/treecmp.html).

### Motif/domain search in Osiris proteins

Using the 23 Osiris protein sequences (Osiris 1 to Osiris 23), we performed the motif search using the Multiple Em for Motif Elicitation (MEME; version 4.6.1 http://meme.nbcr.net) (Bailey *et al.* 2006). To find short motifs, the parameters were set to discover up to 10 motifs ranging from six (minimum width) to 30 (maximum width) amino acids and with any number of repetitions. The 10 motifs discovered covered the two-Cys region, the duf1676 domain, and the AQXLAY domain. The MEME result is available from our website (http://bioinfolab.unl.edu/emlab/Osiris). We used the Motif Alignment and Search Tool (Bailey *et al.* 2009) to search these 10 motifs from all Osiris protein candidates to confirm whether these proteins have the Osiris signature motifs. We also used the Pfam protein family search at http://pfam.sanger.ac.uk (Finn *et al.* 2010) to identify the presence of the duf1676 domain in each candidate. Signal peptide and transmembrane predictions were done by Phobius (version 1.01) (Kall *et al.* 2004). Transmembrane prediction was also confirmed by HMMTOP (version 2.1) (Tusnady and Simon 2001).

### Identification of Osiris homologs

Osiris protein candidates found by blast and profile HMM search using the 24 *D. melanogaster* Osiris protein sequences were identified as Osiris homologs on the basis of the results of 10-motif search using Motif Alignment and Search Tool, duf1676 profile HMM search, as well as reciprocal blast search. For the reciprocal blast, each of the candidate proteins was used as the query and blastp similarity search was performed against the *D. melanogaster* protein set. When none of the known Osiris proteins was found to be significantly similar, this candidate was considered not an Osiris homolog and excluded from the candidate list.

### Classification of *Osiris* orthologous gene groups

Orthologous groups of *Osiris* genes were identified on the basis of phylogenetic clustering as well as the chromosomal location and order of the *Osiris* gene candidates wherever the gene coordinates on contiguous genomic sequences (*e.g.*, supercontigs) were available. We first aligned all Osiris protein sequences and reconstructed a draft phylogeny. On the basis of this phylogeny, preliminary assignment of Osiris groups was performed. Alignments and phylogenies were repeatedly refined for each orthologous group individually. Note that as mentioned previously, alignments were generated group by group as well as using all sequences all at once. We confirmed that the phylogenies reconstructed from two versions of alignments were topologically equivalent, and ortholog-grouping was not biased arbitrarily because of the alignment strategy. When the assignment of a gene to a particular paralog group was unclear as the result of weak similarity (unsupported phylogenetic clustering), a nonconserved location, or both, these were called Osiris-like genes, and are separately listed as such in Table S3. The final results for all *Osiris* genes we identified are listed in Table S1 and Table S3. The sequences and alignments of all Osiris proteins are available from our website (http://bioinfolab.unl.edu/emlab/Osiris). The final maximum likelihood phylogeny is shown in Figure S2.

### Sequence logo of Osiris protein sequences

Using a multiple alignment of the *D. melanogaster* Osiris 1, 2, 5, 6, 7, 8, 9, 11, 12, 14, 16, 18, 19, 20, and 21 protein sequences, the sequence logo was generated using Weblogo 3 (http://weblogo.berkeley.edu/) (Crooks *et al.* 2004). The other paralogs were omitted because they were either missing a domain or have large insertions that make alignments unreliable.

## Results

### Distribution of *Osiris* genes

**Figure 1:**
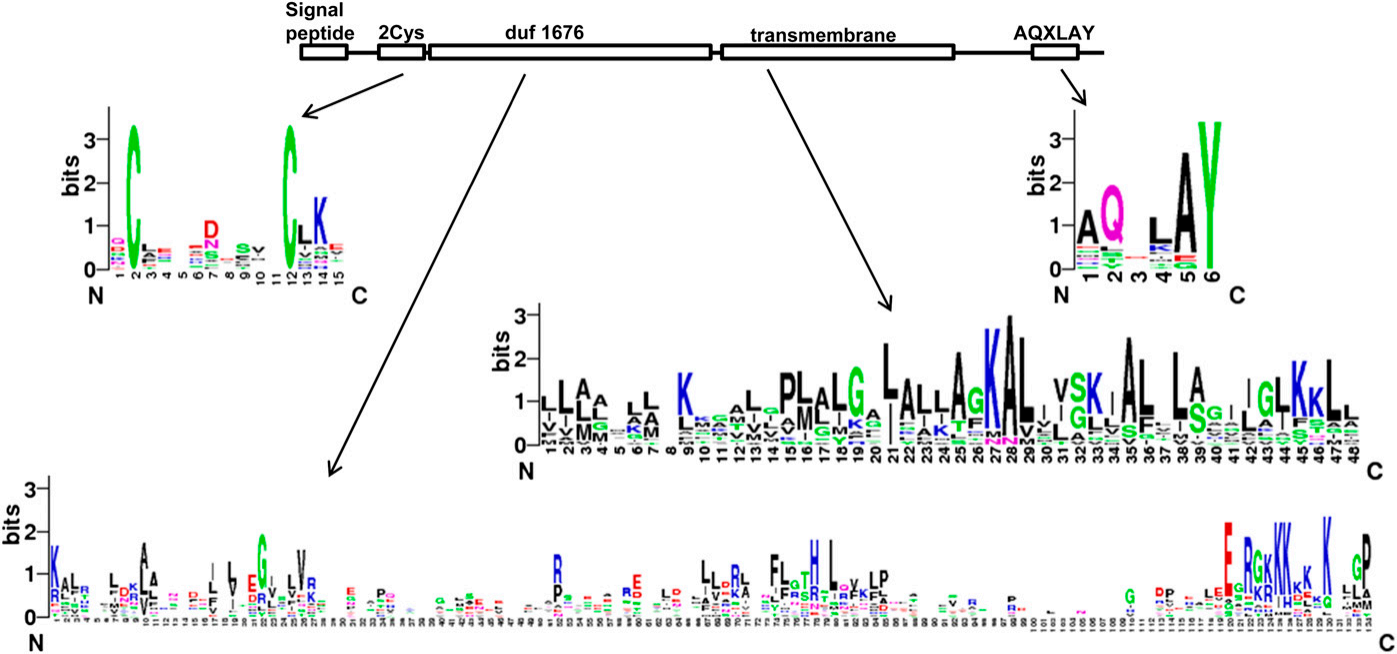
Features of the Osiris proteins. The conserved domains within all Osiris proteins are indicated as boxes. The regions between these domains are conserved only within orthologous groups and serve to identify the different *Osiris* family members. Some examples of conserved patterns for the ortholog-specific domains are shown below with sequence logos.

The accumulation of genomic and EST sequences from a diversity of insects has allowed us to further characterize the *Osiris* gene family. *Osiris* family members are characterized by five features: (1) a hydrophobic region at the N-terminus that is likely a secretion signal peptide; (2) a two-Cys region; (3) a domain of unknown function, duf1676 (Pfam family: PF07898) (Finn *et al.* 2010); (4) a hydrophobic putative transmembrane domain, and (5) a region including an AQXLAY motif and often additional nearby tyrosine residues. These domains are illustrated in Figure 1 (see also the alignment in Figure S1). Although these domains are found in most *Osiris* family members, the regions between these domains are what distinguish family members from each other. The regions between the conserved domains are highly variable, in sequence and in length, but are well-conserved within a group of orthologs from different species.

We identified one family member that had not been previously described in *Drosophila melanogaster*, *CG15589*, which is located between *Osiris 1* and *Osiris 2*. Although the signature domains are not all conserved in the *D. melanogaster* protein (see Figure S1), the orthologs were found to be conserved in other insects as described below. We here rename this gene as *Osiris 24*. With the search strategy and criteria we developed, we did not find any additional *Osiris* genes within the *D. melanogaster* genome.

We searched the NR protein and EST nucleotide sequence databases at NCBI for *Osiris* homologs. The results are summarized in Table S4. *Osiris* homologs were found almost exclusively from insects. Searches using a profile HMM (Durbin *et al.* 1998) for duf1676 against the NR protein database gave us consistent results. Short and weak similarities against two Osiris proteins (Osiris 17 and 24) were found from EST sequences of Collembola (a springtail) and some crustacea (see Table S4 for details). Similarities found in the EST sequences must be viewed with some caution because they can result from contamination of the material used to make the cDNA library. For example, sequences highly similar to insect *Osiris* sequences were found in several plant EST sequences but not in any complete plant genomic sequence, suggesting that they are attributable to insect contamination of the plant material (a footnote in Table S4 provides some specific examples). To determine whether the *Osiris* family is arthropod-specific or insect-specific, we examined the complete genome sequences of two noninsect arthropod species, a crustacean, *Daphnia pulex* (Colbourne *et al.* 2011) and an arachnid, the deer tick *Ixodes scapularis* (Hill and Wikel 2005; Pagel Van Zee *et al.* 2007). Extensive similarity searching using BLAST (Altschul *et al.* 1997; Camacho *et al.* 2009) and profile HMMs showed only weak similarities (lower than the threshold we used) in these noninsect arthropod genomes and none of them had *Osiris* signature motifs.

**Figure 2:**
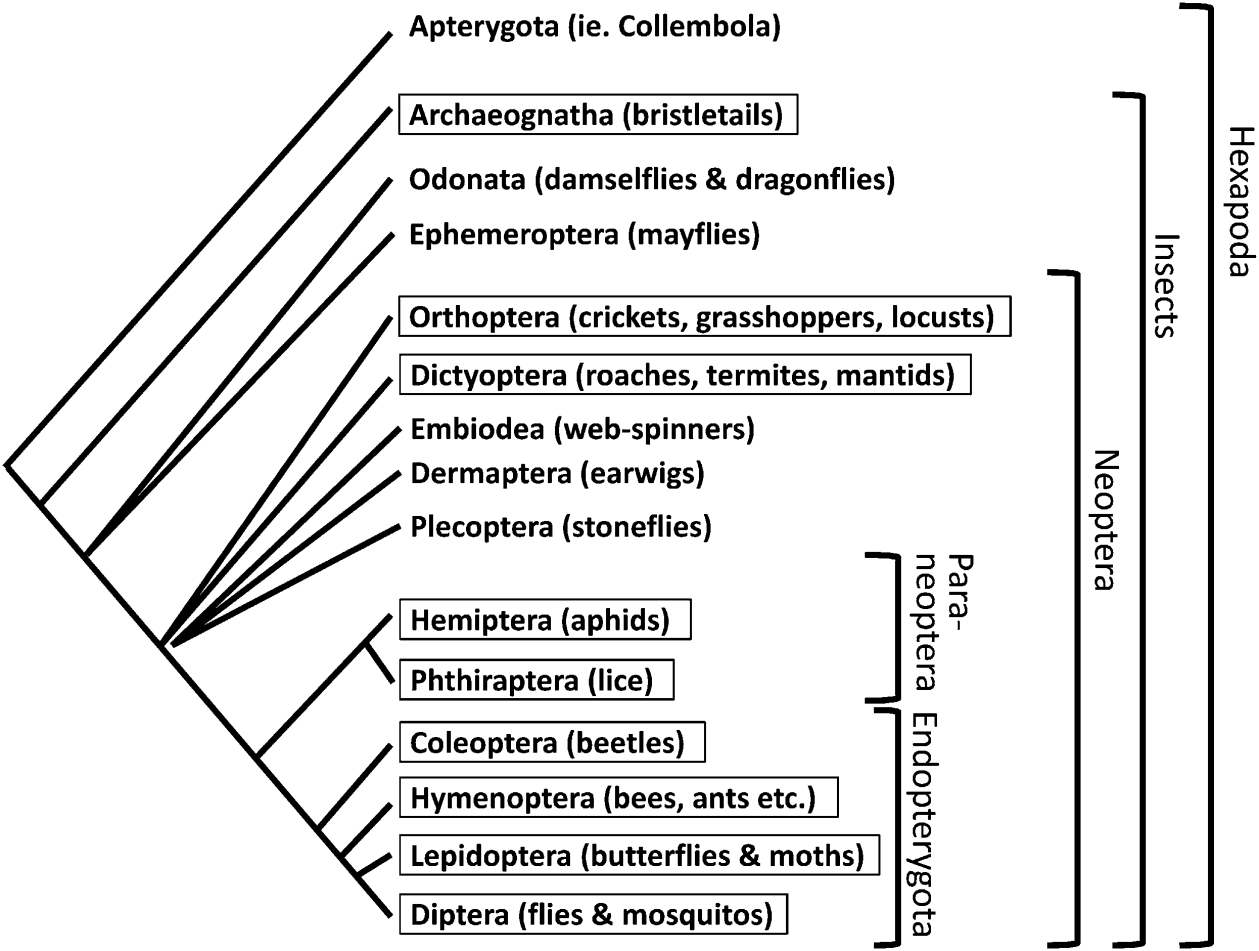
A simplified phylogenetic tree of the insects, indicating the groups shown to have *Osiris* gene homologs. The tree is based on Grimaldi and Engel (2005), Cranston and Gullan (2009), and Whitfield and Kjer (2008). Boxed taxa indicate that at least one *Osiris* family member has definitively been found.

**Figure 3:**
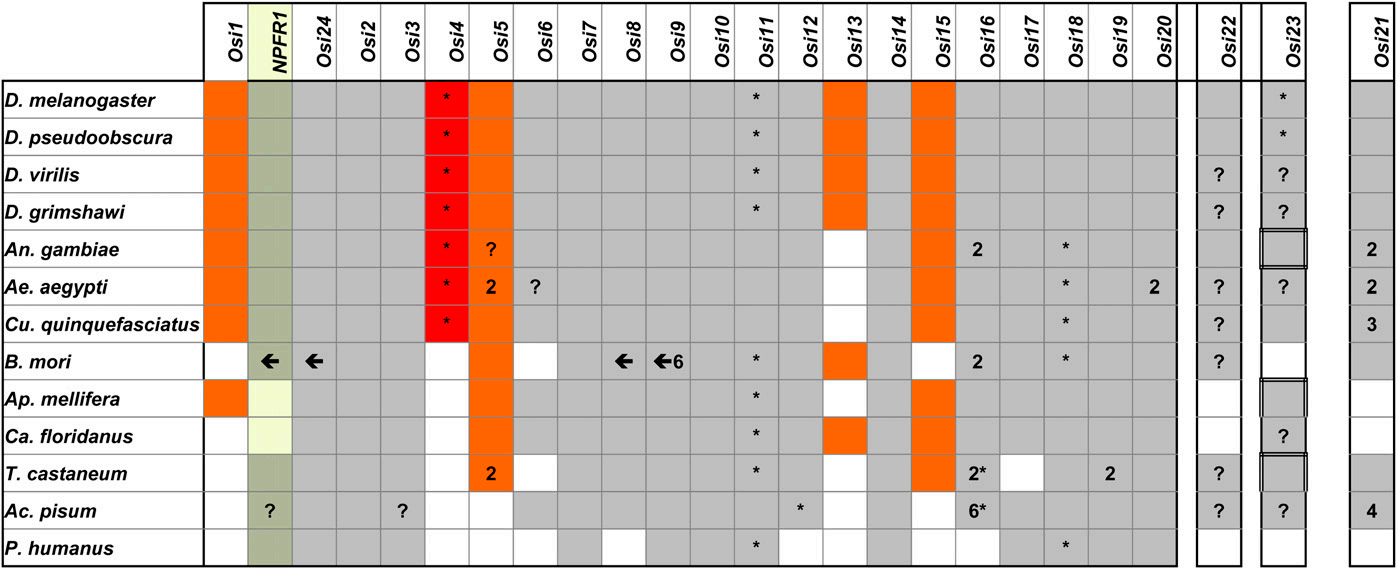
Presence or absence of *Osiris* family members in various species. Gray indicates presence. Diptera and holometabolous insect-specific presence is shown in red and orange colors, respectively. An asterisk indicates that the gene is inverted relative to *Osiris 2* in each species, and a left arrow indicates that the region including more than one gene is inverted. A question mark indicates when the relative gene direction cannot be inferred because of separated contigs. A number indicates the number of duplicated copies. One non-*Osiris* gene, *NPFR1*, is also included in the figure because it is tightly linked within the *Osiris* gene cluster. In the *D. melanogaster* genome, genes from *Osiris 1* to *Osiris 20* are located within a tightly linked 167.5-kb region on the chromosomal arm 3R (83D-E). Although *Osiris 22* and *Osiris 23* are also on the 3R but located distantly (87E and 99F, respectively), *Osiris 21* is located on the chromosomal arm 2L (32E). In some insects, *Osiris 23* is located on a different chromosome and they are indicated by double-line boxes. See Table S3 for detailed information of all genes we identified.

In addition to the genomic sequences from the 12 *Drosophila* species (Clark *et al.* 2007), we searched for *Osiris* gene candidates from the following nine insect complete genomes: three species of mosquito, *Anopheles gambiae*, *Culex quinquefasciatus*, and *Aedes aegypti* (Arensburger *et al.* 2010; Nene *et al.* 2007); the honeybee *Apis mellifera* (Honeybee Genome Sequencing Consortium 2006; Munoz-Torres *et al.* 2011); the Florida carpenter ant *Camponotus floridanus* (Bonasio *et al.* 2010); the red flour beetle *Tribolium castaneum* (Tribolium Genome Sequencing Consortium 2008); the silkmoth *Bombyx mori* (Xia *et al.* 2004); the pea aphid *Acyrthosiphon pisum* (International Aphid Genomics Consortium 2010); and the body louse *Pediculus humana* (Kirkness *et al.* 2010). Figure 2 shows the phylogenetic summary of all insects where we found *Osiris* homolog candidates. As shown in Figure 3 and in Table S1 and Table S3, the majority of the members of the *Osiris* family were identified in these species, indicating that the *Osiris* family was present in the common ancestor of the hemimetabolous and holometabolous insects (paraneoptera and endopterygota, respectively).

EST sequences similar to *Osiris* genes have been found in a number of additional distantly related insects, including the German cockroach *Blattella germanica* (order Blattodea), the cricket *Gryllus bimaculatus* (order Orthoptera), and the termite *Reticulotermes flavipes* (order Isopetera). Twenty-three partial sequences have been found in ESTs from the primitive archaeognathan *Lepismachilis y-signata* (jumping bristletails). These *Lepismachilis* EST sequences were compared using BLAST, and those with significant identities were assembled, resulting in 10 unique sequences. This primitive wingless insect clearly has at least 10 different *Osiris* genes. As Figure S2 shows, all these *Lepismachilis* Osiris sequences form one cluster along with several other unclassified Osiris-like proteins. These sequences may represent ancestral forms of Osiris proteins, and none of the currently available *Lepismachilis Osiris* sequences seems to be closely related to presently known *Osiris* subfamilies.

### Synteny of *Osiris* genes

The synteny of *Osiris* genes (from *Osi1* to *Osi20*) between *D. melanogaster* and *A. gambiae* that was discovered in 2003 (Dorer *et al.* 2003) is largely maintained within the 12 sequenced *Drosophila* species (see Table S1), and with the two additional mosquito species sequenced. Because the *Osiris* gene cluster is so conserved within the genus Drosophila, we have chosen four divergent species (*D. melanogaster*, *D. pseudoobscura*, *D. virilis*, and *D. grimshawi*) as representatives for further analysis.

**Figure 4:**
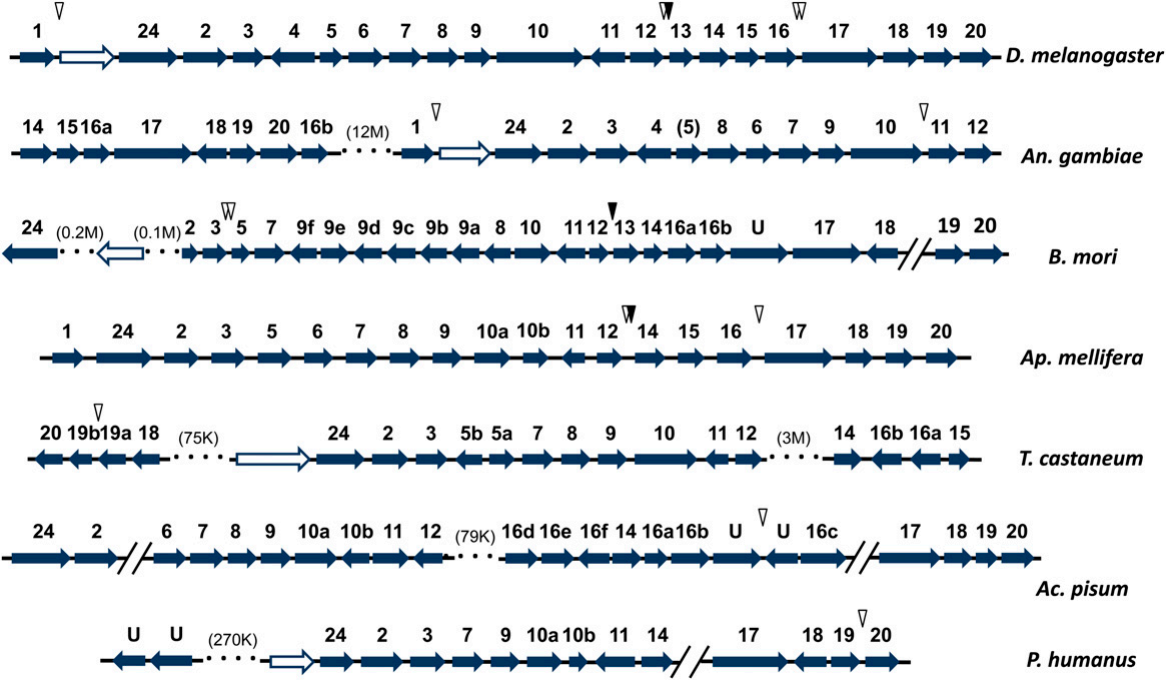
Synteny of the *Osiris* gene cluster. *Osiris* family genes are indicated in their syntenic chromosomal blocks. Paralogs are labeled with numbers, and in the case of duplications with letters, *e.g.*, 9a and 9b. The white arrows indicate the unrelated gene *NPFR1*, which is often in the syntenic block. Slashes indicate unknown linkage because of contig gaps, whereas dotted lines indicate large chromosome distances whose sizes are indicated. “U” means that the identity of that *Osiris* paralog is still undetermined. Inverted triangles indicate inserted non-*Osiris* genes, although some have weak similarities. The filled inverted triangles are similar to the *D. melanogaster* gene *CG15594*. Note that *Osiris 10*s in *Drosophila*, mosquitoes, and *T. castaneum* are twice as long as their homologs in other genomes. In the two hymenoptera, *A. pisum*, and *P. humanus* genomes, two *Osiris 10* genes (*10a* and *10b*) are annotated as individual genes, and in *A. pisum*, *10b* is located on the reverse strand. Therefore, it is likely that having a long single *Osiris 10* is a result of incorrect gene prediction. For our analysis, we divided the longer form of *Osiris 10* into two parts and performed alignments and phylogenetic analyses using them as individual proteins.

The synteny is even more striking when sequences from more distantly related species are examined (Figure 4). Both hymenoptera species, the honeybee *A. mellifera* and the ant *C. floridanus*, maintain almost all of the genes in the same order, and they are each transcribed in the same direction in these two species as they are in fruit flies. There are a few interesting exceptions. A neighboring gene *Neuropeptide F Receptor 1* (*NPFR1*) is missing in these hymenoptera (see Figure 3). *NPFR1*, although it is unrelated to *Osiris* genes, is conserved in synteny with the *Osiris* genes (located between *Osi1* and *Osi24*) in all the insects we examined except for the hymenoptera. *Osiris 4* was also not found in *A. mellifera*, nor any other non-dipteran insects examined, although the neighboring genes *Osiris* 3 and *Osiris* 5 are conserved. Interestingly, *Osiris 4* and *Osiris 11* are transcribed in the opposite direction of all the other genes in *Drosophila* and most other species. *Osiris 1*, *5*, *13*, and *15* are unique to the holometabolous insects we examined. The region between *Osiris 12* and *Osiris 14* is interesting. There are often *Osiris*-like genes, or *Osiris* fragments in this region, but the similarity is so weak that it is impossible to determine their identity. This region is also apparently a region with lower selection on the synteny – this is the location of the inversion breakpoint in *A. gambiae*, and is also the location where other genes have interposed into the cluster, such as *CG15594* and *CG15597* in *D. melanogaster*. Note that some of the signature domains are not well-conserved in *Osiris 4* and *Osiris 13* (see Figure S1), indicating that their functions may be modified in these more derived paralogs.

Although the complete genome sequences from distantly related insects are less complete and have occasional gaps in the scaffolding, the synteny of the *Osiris* genes is conserved over a remarkably wide range of insect orders. Chromosomal rearrangements such as an inversion in *A. gambiae*, and movements of parts of the cluster in other species (such as the apparent separation of *Osiris 2-12* from *Osiris 14-16* in *T. castaneum*), occasionally disrupt the synteny. Gene duplications have also occurred in some lineages, for example, there are multiple copies of *Osiris 9* in *B. mori*.

## Discussion

The *Osiris* gene cluster is a family of genes that is present in all insects but only in insects. Alignment and phylogenetic analysis indicates that the various paralogous members of the gene family each have very distinctive features (see Figure S2). Indeed, the paralogs within *D. melanogaster* are diverged enough to be easily distinguished from one another by the protein sequences between the conserved Osiris domains. The *Osiris* paralogs have also diverged enough at the DNA sequence level that there is no evidence for gene conversion in the recent evolutionary history of the species. The differences between the multiple *Osiris 9* copies in the *Bombyx* lineage (see Figure S2) and the presence of distinct orthologs of the various *Osiris 9* copies in EST collections of other lepidoptera (D.R. Dorer, unpublished data) indicate that these duplication events occurred in the ancestral lepidopteran lineage since its divergence from the hymenoptera and coleoptera and also show no evidence of recent gene conversion. Any given *Osiris* gene is more similar to the orthologs in other species than to any of the paralogs in the same species. Therefore, the common ancestor of all the Endopterygota must have had an *Osiris* gene family cluster very similar to what is seen today in *D. melanogaster*. The presence of at least seven different members of the family in the Archaeognatha suggests that the evolution of this gene family extends back to the time just after the divergence of insects from other arthropods, but before the divergence of the major insect orders from one another, followed by very strong selection ever since to maintain the diverse members of the gene family as distinct entities. Therefore, the *Osiris* gene family must have evolved by gene duplication and divergence events very early in the radiation of insects, perhaps as many as 400 MYA, and has been subject to strong selection in all insects ever since. Minimal available sequence data on non-insect hexapods, such as the Collembola (springtails), and primitive insects such as the Odonata and Ephemeroptera prevents us from further refining the phylogenetic distribution.

There also appears to be very strong selection on the synteny of the gene family, raising the question of whether the synteny has a function. Recent work has suggested that synteny is more common with developmental regulatory genes and maintained due to selection on co-expression (Quijano *et al.* 2008). However, publicly available expression data on Flybase (http://www.flybase.org) indicates the *Osiris* genes are expressed in a variety of tissues, including epidermis, hindgut, foregut, and trachea, suggesting that coexpression is not the selective force. One interesting observation about the tissue expression data is that all of the *Osiris* genes appear to be expressed in tissues derived from ectoderm except for the nervous system. This could be a clue to a specific feature of insect non-neuronal ectoderm compared with other arthropods. The temporal regulation of expression in *D. melanogaster*, although interesting, is probably not sufficiently consistent or unique to explain the conserved synteny either. All of the *Osiris* genes have peaks of expression in one or more of three specific stages of development: 12–18 hr old embryos, second instar larvae, or pupae at 2–3 days post-white-prepupal stage (Gelbart and Emmert 2010). None is expressed to any great extent outside of these three times. However, there is variation within the *Osiris* family, with some being expressed well at all three times, some predominantly in embryos, some predominantly in pupae and at least one predominantly in second instar larvae. This finding indicates that the *Osiris* paralogs maintain their individual functions with some degree of differentiation among them. That the synteny has been conserved so well through such long periods of time, in spite of the high rate of chromosome rearrangement in the genus *Drosophila* (Bhutkar *et al.* 2008; Ranz *et al.* 2001) suggests strong selection on co-localization. In addition, high rearrangement rates were recently shown for coexpressed *Drosophila* genes with short intergene distances (Weber and Hurst 2011), making the synteny of the *Osiris* gene cluster even more remarkable.

As mentioned previously, the *Osiris* signature domains are highly conserved among the majority of the *Osiris* family members. The exceptions are the aforementioned *Osiris 4* and *Osiris 13*. *Osiris* genes that are not located in the syntenic cluster (*Osiris 21*, *Osiris 22*, and *Osiris 23*) also have more weakly conserved signature domains (Figure S1). This implies again the possible association between the co-localization of *Osiris* genes and their functions.

The *Osiris* gene family was an early evolutionary innovation in the divergence and spread of insects. Its conserved domains are unique. The extreme dosage effects of the cluster in *Drosophila melanogaster* may be part of the explanation for the selection on synteny. Clearly the *Osiris* gene family is in need of further study.

## Supplementary Material

Supporting Information
