# Supplementary material for: Evolution of a Large, Conserved, and Syntenic Gene Family in Insects
Source: G3 (Bethesda). 2012 Feb 1;2(2):313–9. doi: 10.1534/g3.111.001412 (PMC3284338; doi:10.1534/g3.111.001412)
Supplement: Supporting Information [file supp_2_2_313__index.html]

Supporting Information 

# Evolution of a Large, Conserved, and Syntenic Gene Family in Insects

## Supporting Information for Shah *et al.*, 2012

**Files in this Data Supplement:**

- Supporting Information - Figure S1 and S2 and Tables S1-S4 (PDF, 2.5 MB)
- Figure S1 - Alignment of *D. melanogaster* Osiris protein sequences (PDF, 110 KB)
- Figure S2 - The maximum likelihood phylogeny of Osiris proteins reconstructed by FastTree (PDF, 1.1 MB)
- Table S1 - Organisms found to have sequences similar to Osiris proteins (PDF, 85 KB)
- Table S2 - List of all *Osiris* genes identified from the 13 complete insect genomes (PDF, 110 KB)
- Table S3 - The list of 24 *Osiris* genes in the *D. melanogaster* genome and those identified from the 11 other *Drosophila* genomes (PDF, 98 KB)
- Table S4 - The list of data sources for the arthropod genomes used in this study (PDF, 74 KB)
