# Supplementary material for: Evolution of a Large, Conserved, and Syntenic Gene Family in Insects
Source: G3 (Bethesda). 2012 Feb 1;2(2):313–9. doi: 10.1534/g3.111.001412 (PMC3284338; doi:10.1534/g3.111.001412)
Supplement: Supporting Information [file supp_2.2.313_FigureS1.pdf]

```
1_CG15585     .. CR----VVLAL---LLL--VLLHY-----CQAKDEGETTQRGAVLVSLT .. -----------ENMAKSAGSQL-V--QADPFISRNQKQC-------------------FET-RSLVSCIKYKT
2_CG1148      .. --MAMRALIFL---ALA------------TLVAGEGLRLPDQQSSNNIQ .. ------------------------GNDNDPFLARTNSNCL--------------------G-GDLSECFKTQA
3_CG1150      .. QTFKVCALLAF---CFV-LVSAR-------------------------- .. -----GSKRRDGTVTISESERK-N--IEDFLLAKLKQNCR------------------QED---DRACKMVKM
4_CG10303     .. HLVASCLLLAL---G-L--NMSLA-----AIHKRSGANS---LGVD--- .. -----------GKPA-ANPAVS-V--ENTDLLDKLSWKC-----------------------ANNASCLYGVA
5_CG15590     .. ------MFRTF-PLLCL--LFLT-------------------------- .. --------------------------------AVRSENC-------------------DQDAGATLYCRGERA
6_CG1151      .. FVATACILLLA-AGISADPVKAA-------------------------- .. -----------------------------EEQPGAFAQCL------------------ESD---SISCLQLTL
7_CG1153      .. MASHKVTFGVL---CLV--ALSAA------------------------- .. --------LPAEETRGHARNAI-G--GENDIMDSIYSDCL------------------RKD---SVSCVKYKL
8_CG15591     .. ------MIKYV---WHV--AALMIVFCWLSSARSASYQH---------- .. ------------SNPGMGSTGL-W--KDMSMVYRIYQQCS------------------GDN---MSVCLKVKL
9_CG15592     .. ----MFKFVCL---FAL-IASTA-------------------------- .. ----------------AATSEA-D--SLLTSALKMVKDCG------------------ERS---MVLCMKERA
10a_CG15593   .. -------MSPL-DIVLL--LVSF------HQVIGSDFSAMSLE------ .. -----------------------------------FKQCVRG----------------SQK-PKIGECLGRSA
10b_CG15593   .. ------------------------------------------------- .. -------------------------------------------------------------------CLGSES
11_CG15596    .. --MESRLGLLI---ALL-LAAFQA------------------------- .. -----WAMEA--PSNYNQNSTE-S--GLLRTVRHIYGQC-------------------AYS-EDVFWCCKIQG
12_CG1154     .. KQWPWQ--ALI---SLLSVLFLA-------------------------- .. CGAATEQL----GSPPGPSPSQ-S--AGARTLLRVYDECT------------------RAE-AGFVPCLKKKA
13_CG15595    .. FKSSISIVVLH---LLL--LVSFG-----GAYTL--------------- .. -------------------------------------------------------------------------
14_CG1155     .. ----MKVFAIA-CVTLL-AASCV-------------------------- .. ------------FAAPSVQDNQVEGDNTLGRAARYLGAC-------------------LES-DDMATCLAVKG
15_CG1157     .. ----MCAKFVC---VVL--LASLV-----C-GSMALPSQDNT------- .. ---------------------------------------------------------------------ERDL
16_CG31561    .. ----MLLTKTV-KYLFY-LALFA-------------------------- .. FMCKYATAASVQPTEVP-AVAPETIRIPQRA-ESLLSGC-------------------EAS-SFSWMCLKIEF
17_CG15598    .. FLL--LICLIN---SAA----KAD-----GTARN----GRLGRHLSTTP .. -----------PT----RKPMD-K--MAPSDSLLLRLARR----FASGNELWDGLVRDCYL-KPDVSCFQKNV
18_CG1169     .. ------MKSTA-ACLIV--ALAAL-----STAHSA-------------- .. ------------PTEGQV--AP-Q--SATQLALDMYHGC-------------------L-K-DLSVSCVRPKA
19_CG15189    .. ---------MAKLLLVIVGVAAL-------------------------- .. -----------VAAGQAAGGSTE---KMQRLIAEEQNKC-------------------ASG-QDSMACIKERA
20_CG15188    .. AFR---STSLL---AFG--CALLL-----VASTSVSGAAIENA------ .. --------------V---TPRI-H--SSDELISTIVDKCF------------------H---ANAMHCLKEKV
21_CG14925    .. ----KRLEWLL---LLA--LVASV-----STAVTPRRRRHSAVESA--- .. ------------PGDWGTAWGL-G--PEMALVRRVYDDC-------------------QDK-NDFIGCLKQKA
22_CG8644     .. -----MSDLVR---FLL--LSVLC-----SSLALAQASSSDG------- .. -----------NQETST---VS-Q--EAARGLA-SSYEPEDKQALRKNSHIFMGIYKNYKS-TYLGNKTTSEY
23_CG15538    .. R---VPSVLVT---FLL--GVILV-----DRYAAGEDLADK-------- .. --------------------SW-I--SQMKKLRSDLRDCYQSGIHQS-----------------LWSCFRSRS
24_CG15589    .. NSHRKRRCSPL---LYG--IILLC-----KVAMIPAAVPE-SELDAGAL .. S----------PRKQESESSAA-A--FK-------------------N------QTITSI-DAELKGLEDLL

1_CG15585     S-KLIWKLA---TNSMGFFPSEYGRDLAGD------------RGRWLRLVQL----------------------------------------------GEPADEVVVFNDAKSLEGDSELTM-------
2_CG1148      L-NTFDEIFF---------------KDQYK------------LSDFARVVRLPE----------TQQR--SLLQEPF---------------------------------------EYSEEPRGDDDEW
3_CG1150      S-IVMNHLY--L--------------NTRID------------LGDRFKVTENGN----------ISMV-----------------------------PDDPEVNQLLSRSM-----GSDEET-------
4_CG10303     N-GLMASYRR---------------GETLK----------LGLFDLVKLPELDA----------SRKHKW----GTGRG--------------------------------------------------
5_CG15590     LRNVLRNLN--RS------------DKPLV------------VIRGLEIVPLQN----------NSIS------------------------------------------------------------D
6_CG1151      F-RKAKSVF---D-------------NPQIE------------LFGGVSLVKSNE----------GRQG----------------------------KSLDNSLAVEAAPTV-----EARTAE-------
7_CG1153      F-SFVDKVL--GA------------RDQFA------------LTEGVTVVRSPD----------APQQ-------------------------------------EAARSI------SGDES-------
8_CG15591     L-TGLEKAF--RS------------AKSLS------------LMEGIQFVSSGG----------ESEE------------------------TKRAPISEKDIEAVLPRSV-----DAKEQV-------
9_CG15592     L-HYFDAE-----------------NGDVR------------LTEGIALVKTDE----------IPVG-------------------------------RSLNEMQLPEEV-----EAREAE-------
10a_CG15593   L-NFIQKLD---E-------------SDNVK------------FVEDFVTVKSET----------AAVR-----------SLNSVLDTDPVDF-------------------------------------
10b_CG15593   E-QLLDGAT--RD------------NSTWQ------------ITDYLSIEPK---------------V--GISKPE----------------------------------------TRRMD--------
11_CG15596    V-RLLGRAL--K-------------VPQLG------------IVDGVSLVRR------------ESFT------------------QDTRSGRSSLLESQ-LSNRDLE--------HLSGKS-------
12_CG1154     I-SFIDRLA--P-------------IDAIN------------VAEGIKLVRLET----------APRP---------------------------PATSENELESSLPRSG-----SDRDAK-------
13_CG15595    -------------------------PPVVQ------------MGGAIVAAVEQD----------AEQE--AAAEER-----------------------------------------QRVERHWLSMAE
14_CG1155     I-TALNRAA--R-------------SNNIE------------LASGVTFQRDPA----------SPVS------------------------RTGKSMSEQDVYAELPQNA-----DERTGR-------
15_CG1157     V-NMLNRLD---S-------------EESVA------------LFGGLRIDRSES----------GRSF--GASKAV-----------------------------------------------------
16_CG31561    V-KIMEKLA---E-------------QEELN------------VLPGISVVKDEN----------A-----TELKTS-------------------------ELMAEVARSYP----SDPSTR-------
17_CG15598    F-SYLDNVLDV---------------QDVN------------VTQRLKFFKNQVDYQVDKEKEEH----------SEARAA--------SAETPIEE--------------------------------
18_CG1169     L-QWFNSAL---R-------------QPEVR------------ITERLSIVRTAE----------KVE-----------------------------------------------------------SRS
19_CG15189    M-RFVDNVM---S-------------KDSFQ------------VSN-LEVRSNGE----------KTTPINEARAS------------------------------------------------------
20_CG15188    L-TYLDTVA--NV------------EEEVS----------GRA----LGDDV-----------------------------------------------------------------------------
21_CG14925    L-HALSRAL---D-------------QDSIK------------IVDGLALEKQNQ------------------SETESILG------------------------------------SLTDARQFG--NL
22_CG8644     KKRLRDRVSA----------PQMAENETPEMVEPDQEDQRDSLAQEIRQDAQAE----------ALME--TQTESPNYDDSESLAAKKRRKRKRKDRNKRKDE-------V-------------ESETD
23_CG15538    L-HIFEGIM---S-------------SPEIS------------IYDGVRLVAA-----------------------------------------P----NS---TD-NATRPDDERKDLKHLT-------
24_CG15589    V-DYVEQFFS---------------NGRYE------------PTPGLVLALQQN----------HSHP--QSYTG--------------------KR--------------------------------
```

```
1_CG15585     ----ILKFL-----KRAMETFGRNH-GLQLRLN---SEGGARVMEES------------------ .. E-----AR- .. -----------LK-RKKKK---WLI----ILPLVILMKIA--HLK--
2_CG1148      --NQLLKYG-----LRRAERFIKST-ALEVEWP---E-ELTEAGRYEARFIGNDIDGELDLIDDG .. QRAGHFSR- .. ------------K-KL-KK-----M----IIPLLLVLKIF-KLKL--
3_CG1150      ----FALLM-----ANKLWKFIRSR-SLRYKFS---E-NTDFVI----NSDPEGSLNLGVSVRPL .. E-----GR- .. -------------G-KM--K-----N----MGPLLMMMAAK----T--
4_CG10303     --------------LSGFMDFVTEN-AIRVPVG---P-MVFSVQRAEDDSDYIEVALLKKTSSST .. ------GRL .. G--RRRHQH-QDK-KQ-FQ-----M----FIPMYLAATTF-------
5_CG15590     EEPDQEQGL-----LDSLSFYLRTH-EINVKLA---D-LLEDES---QVS--------------- .. E-----AR- .. ---------------KK-DK-----G----QGMLLAMALMF----G--
6_CG1151      ----MGNYF-----MDNAKSFFAER-SLNFNFA-----------NAARSVARAIPDDIKADLREL .. E-----SR- .. ---------TRKK-KL-LK-----K----FLPILLGVGAK----I--
7_CG1153      ----FESLA-----LNRISSFLNSH-TIKVELK---G-ADIVQA--VSSTGR------------- .. E-----SR- .. ---------GKKK-KA-AK-----I----LGPILALVALK----A--
8_CG15591     ----LNNMI-----LKRVGNFLQDH-TLQVKFDN----EANSV---------------------- .. E-----GR- .. ------------K-KKEKK-----G----NGAMIMIPLLL----G--
9_CG15592     ----VDSLL-----VERVARFFGTH-TLQFKVP---K-------DSIQDMQRALE---------- .. E-----SR- .. ---------GKKK-EK-KK-----Y----LMPLLMLFKLK----M--
10a_CG15593   ------RGI-----LENAGAVMGQR-SMEWHMD-------------------------------- .. T-----GR- .. ------------V-LT-KQ-----Y----LLPFLLGLKFN----L--
10b_CG15593   ------MGL-----PGKLLELVQGR-ALRLQLP---R-QLTISNAIDDFGSELGLD--------- .. Q-----GR- .. ------------K-KK-DK-----D----KNMAMMGGMIM----M--
11_CG15596    ----LDALL-----LERFLNFVHSH-QLQVNLP-----RLLRFGE--RNVQDWLLHVVGYFMPAS .. E-----GR- .. ------------KKKDDKK-----Y----LGPFIAAVLLK-------
12_CG1154     ----LTNML-----IERLSYFFNGH-SLQVSFP---K-------LTSDEIGRGLE---------- .. E-----GR- .. ------------G-KM-KK-----M----MGMMMMGMAMK----M--
13_CG15595    --TQLHSLITDDLSTEEVNNML------------------------------------------- .. ETWSTEGR- .. ---GKH---KKQK-KL-MK-----M----VYPLLAAVAVA----K--
14_CG1155     ----LVDLA-----VSSAADFLSTH-NLEFKLP---A-------ETTQQVARALD---------- .. E-----GR- .. ------------G-KI-KK-----M----LGPVALAIGAK----L--
15_CG1157     ------ESF-----EDRAERYLETH-ELNLSFS---G-DEQDENSENEYTGRAMD---------- .. E-----SR- .. -----------SK-RM-KK-----M----LLPLLLALKLK----K--
16_CG31561    ----LNGYI-----VAKLENLLRTR-FLRFRLL---D-DKSLV---------------------- .. E-----GR- .. -------KHKFGK-KG---------G----LEALVAAGVMM----K--
17_CG15598    ----VTSAL-----YGKSIKFAMTHDLEVDLPE-----------------------------VM- .. ALLLIIKI- .. -----------------IK-----IKLFWLLPIVIGVGAAKKLLLKF
18_CG1169     --MNPEERL-----FDDIDSYLGSH-SLRIQAP---E-YFRTSE-ARSLVPDFLMSNPLTQGGLV .. E-----GR- .. ------------G-MI-RK-----A----VLPFLLGLKLK----T--
19_CG15189    ----SADGF-----LDAIENYIRGH-DVSMDLP---L-ADAKVTVSARNLVNNQLSLNLQLNGDD .. E-----AR- .. GKKGNIFKKGKKH-RL-RK-----L----AMPILVLILLK----A--
20_CG15188    ----IDKVI-----VDRLGRILNTN-EMRLQLP---Q-TFFAGSVVTYRSDRG---FDLE-LPKD .. E-----KK- .. ---------------K-DK-----L----FLPLLLLMKFK----L--
21_CG14925    --SPIDRAL-----LSKADKLMRTH-TLKIDMDVGGGEDS----VGR------------------ .. ------EH- .. ------------GHKK-KKHKEGGH----IKYVVAALLTA----M--
22_CG8644     --QPDPAPE-----DETIQRYNVGP-GLNVSLD--MS-NDIVHVKLDGENLKEIIGARWLTLDNS .. E-----GRG .. -----------------KKYDMITK----VLPLFILPFLI----Q--
23_CG15538    ----WFDQL-----AVSLAKGLTTH-TLQVNLG---K-LTERYLSSDP-------VG-------- .. S-----AR- .. -----------RR-HR-YN-----M----IITMMFGVTAL----G--
24_CG15589    ----TTRSI-----LE------------------------------------------------- .. RLLFFSGL- .. -----------------KK-----V----MWPIYMGLQVLKSVLF--

1_CG15585     MTLVSMLMG---VLGMNVL . ------ . LVGGVGWLIHYLKYKTMCKIHP---------------------------------------- .. WATSKAYNA-HNYLDTISKRIQ------ ..
2_CG1148      LLFLPFILGIAGLKK---- . ------ . ILGLAAIVLPGL-----FAYFK---------------LCRPPGGVGGA-FGGGLSGLFGGKNT .. -PQEAAYNGYYGRNSGKDIVAEQQPQKS ..
3_CG1150      GMVGALLLKGLFLLAGKAL . ------ . IVSKIALLLAVI-----ISLKK---------------LL-S---SK----------------- .. -AQNMAYSG-QQPGKVAQ---------- ..
4_CG10303     -GWTMVAAKAVGLLTLKAL . ------ . ILSKIAFVVAAI-----VLIKK---------------LMDN--------------------- .. HM--PYYRS------------------ ..
5_CG15590     KMMAVMGLGGIAALAMKAL . ------ . GVSLVALMMAGM-----LGLKT---------------AA-Q---HG---------------- .. ----LAYRG-WD--------------- ..
6_CG1151      AVLGVGSIFGLLFLAKKAL . ------ . VVSVIAFFLALA-----AGASS---------------GLGRIGGSGG-GGGLLGGLGGLFGGK .. -AQTIAYQG-YKQARR----------- ..
7_CG1153      AALLPLLLGAIALIAGKAL . ------ . LIGKIALVLSAV-----IGLKK---------------LL-S---QE---------------- .. -AQDLAYGA-QKPVQA----------- ..
8_CG15591     GTIVPLAYGALAMLAGKAL . ------ . IVSKLALVLASI-----IGIKK---------------LL-S---GGGG-G------------ .. -ELDTAYSG-WKPAAKESAGSAKSL--- ..
9_CG15592     AALLPLAIGFLALISFKAL . ------ . VIGKIALLLSGI-----IGLKK---------------LL-E---SK---------------- .. -AQQLAYAA-YKQ-------------- ..
10a_CG15593   VALVPLIFAGICLLLKKSL . ------ . FLVKLAIYVSSF-----LGLGG---------------IVGGL-----------------GGLG .. ----TVFGKGQDEFHH---------QYD ..
10b_CG15593   ATLAQMFLGKVILIAGSAF . ------ . IMAKIALVISLL-----GSLKK---------------GS-T---GHS--------------- .. GSHMEYYQA-YQMEPLKRR-------- ..
11_CG15596    TAILKMAYHSIAIVAGKAL . ------ . IVGKIALIISAI-----IGLKK---------------LV-G---HDGG-------------- .. -MQDKAYQA-WMPHVAASPSPVAKGS-- ..
12_CG1154     MGMIPIAMGALYILAGKAL . ------ . IISKIALLLAGI-----IGLKK---------------LM-S---GKSS-GGSSGWSS------ .. -AQELAYRA-HHQEQVAHAQSRPQ---- ..
13_CG15595    VVLLPLILKWLTALSTSSF . ------ . VMGKIALVTSGI-----LALKW---------------ILSGGHAHDRL-E------------ .. ----IIHSH--APLVKGLH---ASDLSS ..
14_CG1155     FAVIPLVLGFLALLTFKAV . ------ . IVAKLAFFLAIL-----VGGSR---------------LL-G---GFGN-KFGG---NSFAGAY .. -AQQLAYAG-QQQQ-------------- ..
15_CG1157     AVVVKIMFTIIKFISLKAL . ------ . AISFLALILAGA-----TFFKD---------------LL-A---KKK--------------- .. -AADLAYNH-YGLAQPF---------- ..
16_CG31561    GMLMAMGLGAIALMAGKAL . ------ . MTALMALTLSGV-----LGLKS---------------LA-G---GGG---------------- .. ----FGYGG-YARSLKVDQSANKI---- ..
17_CG15598    LLFLFPALSHLFKLCSHYQ . ------ . ILLKIDKIIEQL----------GVKN--DLCKERIVCSMYKDPATYSP-HSNFISAELSRDTS .. ----YRLIQAARDGQDQKDCQSLYPQCN ..
18_CG1169     TVLVPLALGLIALKTWKAM . ------ . TLGLLSLVLSGA-----LVIFK---------------IAKP--------------------- .. -AQDLAYAGQK---------------- ..
19_CG15189    ITVIPMAIGILKIKAFNAL . ------ . ALGFFSFIVSVG-----LAIFQ---------------LCKK--------------------- .. LGQALAYQA-YA--------------- ..
20_CG15188    KVIMPILLALIGLKATKAL . ------ . ILSKIAIKLVLG-----FLIYN---------------LI-Q---KLG---GMK--MNMVPMPA .. -SQNLAYSS-YHPSSSSSYSSGSSSGSS ..
21_CG14925    GIAGPLGLKALAAIAGKAL . ------ . VISKVALTIAGI-----IALKK---------------LFSH--------------------- .. ----DPYRYYYEYHQQ----------- ..
22_CG8644     SAIVPFLVTKLKLLLVKSI . ------ . LVGKLAIFLLII-----SAIKN----------------------------------------- .. SSAAAAYNG-YRVEGKPTTWIS------ ..
23_CG15538    AILVPMGFQMLSIVSGKAL . ------ . LLAKMALLLASI-----NGLKR---------------VA----------------------- .. -NNGLHYGLYHVPGE---HLGGY-YDRG ..
24_CG15589    AMFLPTIISSVSRLIGKGI . ATLSRY . LLSRLDSVFAQL----------KLNPENEACREKLICLMYANPAKYAP-YSNLVSAQLSRELN .. ----FKYMRAAKDGQDGVDCDESFAKCK ..
```

Figure S1 Alignment of *D. melanogaster* Osiris protein sequences. Multiple alignment was generated using the entire Osiris sequences and aligned *D. melanogaster* sequences are extracted and presented above. The five Osiris signatures are color-coded: predicted signal peptide in red, 2-Cys region in orange, duf1676 region in blue, predicted transmembrane

region in green, and AQXLAY motif in purple. Long insertion regions are excluded and indicated by "**..**". Osiris 10 proteins are divided into two parts, and included in the alignment as 10a and 10b.
