# Supplementary material for: Evolution of a Large, Conserved, and Syntenic Gene Family in Insects
Source: G3 (Bethesda). 2012 Feb 1;2(2):313–9. doi: 10.1534/g3.111.001412 (PMC3284338; doi:10.1534/g3.111.001412)
Supplement: Supporting Information [file supp_2.2.313_FigureS2.pdf]

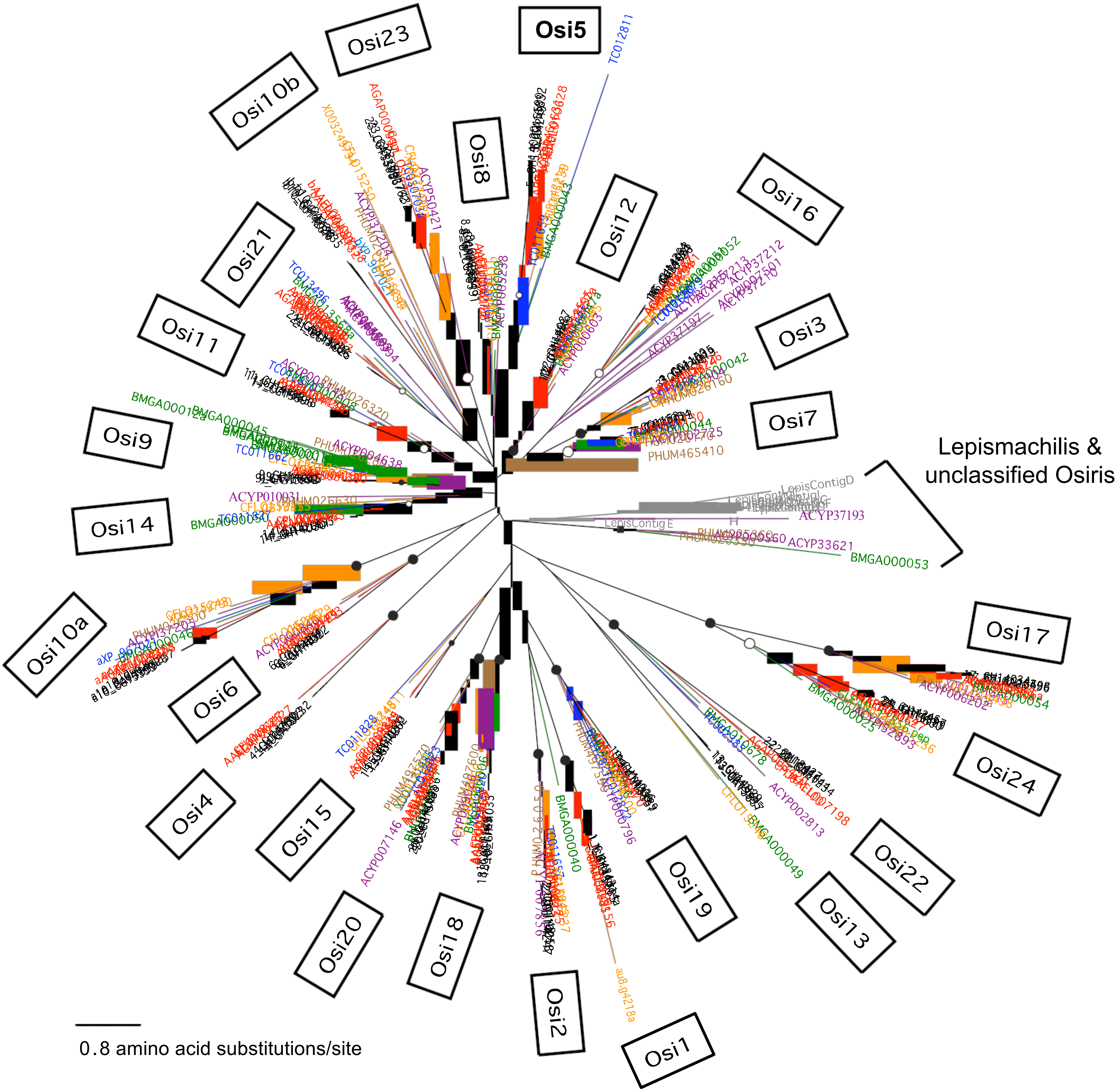

Figure S2 The maximum likelihood phylogeny of Osiris proteins reconstructed by FastTree. See Supplementary Table S2 for the sequences included in this phylogeny. Sequence labels and branches are color- coded based on species: four *Drosophila* species in black, three mosquito species in red, *B. mori* in green, two hymenoptera species in orange, *T. castaneum* in blue, *A. pisum* in purple, and *P. humanus* in brown. Ten sequences assembled from *Lepismachilis y-signata* ESTs are also included and shown in grey. Osiris 10 sequences were divided into two parts and aligned individually. These sequences are shown as “Osi10a” and “Osi10b” above. Major clusters supported by higher than 90% or 70% bootstrap supporting values are shown with solid and open circles, respectively. Different sizes of the circles are used for the supported clusters that include all species in the group (large circles), only holometabolous insects (middle circles), or only dipteran species (small circles).
