# Supplementary material for: Evolution of a Large, Conserved, and Syntenic Gene Family in Insects
Source: G3 (Bethesda). 2012 Feb 1;2(2):313–9. doi: 10.1534/g3.111.001412 (PMC3284338; doi:10.1534/g3.111.001412)
Supplement: Supporting Information [file supp_2.2.313_TableS1.pdf]

Table S1 Organisms found to have sequences similar to Osiris proteins.[a, b]

**[Phylum] Subphylum; Class** [Arthropoda]	**Superorder**	**Order**	**Number of species and list of genera**	
Hexapoda; Insecta				
Dicondylia; Pterygota; Neoptera				
	Endopterygota	Diptera	25	*Drosophila, Aedes, Anopheles, Culex, Cochliomyia, Glossina, Haematobia, Lucilla, Phlebotomus, Polypedilum, Simulium, Sitodiplosis, Teleopsis*
		Lepidoptera	16	*Bicyclus, Danaus, Heliconius, Choristoneura, Tineola, Papilio, Bombyx, Antheraea, Manduca, Spodoptera, Mamestra, Heliothis, Trichoplusia, Ostrinia*
		Hymenoptera	9	*Nasonia, Bombus, Apis, Megachile, Acromyrmex, Solenopsis, Camponotus, Harpegnathos*
		Coleoptera	5	*Callosobruchus, Dendroctonus, Tribolium, Diabrotica, Onthophagus*
	Paraneoptera	Hemiptera	11	*Acyrthosiphon, Adelphocoris, Aphis, Diaphorina, Homalodisca, Myzus, Maconellicoccus, Nilaparvata, Peregrinus, Rhopalosiphum, Toxoptera*
		Phthiraptera	1	*Pediculus*
	Dictyoptera	Blattodea	1	*Blattella*
		Isoptera	2	*Reticulitermes, Coptotermes*
	Orthopterida	Orthoptera	3	*Schistocerca, Locusta, Gryllus*
Monocondylia		Archaeognatha	1	*Lepismachilis*
Hexapoda; Entognatha				
Collembola		Entomobryomorpha	1	*Folsomia*[c]
Crustacea				
Malacostraca		Decapoda	2	*Penaeus*[c], *Homarus*[c]
Branchiopoda		Cladocera	2	*Daphnia*[c]
[Platyhelminthes]			1	*Clonorchis*[d]

[a]Search was done using blastp and tblastn using each of *D. melanogaster* Osiris protein sequences as queries against the non-redundant protein and EST databases at NCBI. The E-value threshold is 0.01.

[b]Highly similar sequences were identified from several plant EST and cDNA sequences. However, these sequences are not included in this table. Such similarities are not found from any complete plant genomic sequences suggesting insect contamination of plant materials. Examples of these cases include: CX523685.1 (an EST from *Medicago truncatula*) with higher than 95% protein similarity ($E\sim10^{-66}$) against *Drosophila* Osi7, and BT086177.1 (a complete cDNA from *Zea mays*) with 64% protein similarity ($E\sim10^{-69}$) against aphid Osi6.

[c]Weakly similar EST sequences were found from Collemboa ($E=\sim10^{-4}$) and Crustacea ($E=10^{-18}\sim10^{-4}$). Reciprocal blast search indicated possible homologous relationships with *Drosophila* Osi17 and Osi24.

[d]One Platyhelminthes EST sequence (FS162944) had high protein similarities against *Drosophila* Osi7 (84%, $E\sim10^{-46}$).
