# Supplementary material for: Evolution of a Large, Conserved, and Syntenic Gene Family in Insects
Source: G3 (Bethesda). 2012 Feb 1;2(2):313–9. doi: 10.1534/g3.111.001412 (PMC3284338; doi:10.1534/g3.111.001412)
Supplement: Supporting Information [file supp_2.2.313_TableS2.pdf]

Table S2 List of all *Osiris* genes identified from the 13 complete insect genomes.

**ID:**	All IDs are for the first transcript/peptide (-RA/-PA) unless noted otherwise
**Direct:**	1/-1 show the direction relative to Osi2 (1 as the same direction).
**Start/End:**	Start and end positions for the coding sequences. For *A. pisum* , transcript start/end positions are shown. When genes are not on the same chromosome or supercontig, only the strand information (+/-) is shown.
**Color codes:**	(orange) annotation changed by us in this report

(Osiris group assignment)

no color	based on both synteny and similarity
(green)	based on synteny but similarity not conclusive

Table S2 List of all *Osiris* genes identified from the 13 complete insect genomes.

	Osiris 1							
**Genome**	**ID**	**Length (AA)**	**Chromosome (linkage group)**	**Direct**	**Start**	**End**	**# exons**	**Note**
D. melanogaster	CG15585	308	3R	1	1999367	2000555	3	
D. pseudoobscura	GA13829	304	2	1	15819977	15818610	3	GA13829-PB (FBpp0298266)
D. virilis	GJ14243	323	scaffold_12822	1	1897146	1895783	3	
D. grimshawi	GD14014a	307	scaffold_14624	1	2876740	2875141	3	based on the last 2 exons of GH14014 GeneWise+Augustus model 2875141..2875544,2875805..2875927,2876344..2876740
An. gambiae	AGAP004121	284	2R	1	50327269	50328282	3	
Ae. aegypti	AAEL015156	282	supercont1.1530	1	22088	5471	3	
Cu. quinquefasciatu	CPIJ006985	293	Supercontig3.144	1	369648	358007	3	
B. mori	None							
Ap. mellifera	au8.g4218a	276	Group15.14	1	546442	548486	4	Based on au8.g4218 ex1-ex4: (546442..546663, 547241..547508,547660..547920,548407..548486) Similarity is weak, no signature domains; but cluster on
Ca. floridanus	None							
T. castaneum	None							
Ac. pisum	None							
P. humanus	None							

	NPFR1							
**Genome**	**ID**	**Length (AA)**	**Chromosome (linkage group)**	**Direct**	**Start**	**End**	**# exons**	**Note**
D. melanogaster	CG1147	485	3R	1	2014920	2019871	4	4 isoforms, all identical aa seqs
D. pseudoobscura	GA11019	498	2	1	15808917	15807000	3	
D. virilis	GJ14245	516	scaffold_12822	1	1885599	1883380	3	
D. grimshawi	GH14016	511	scaffold_14624	1	2866563	2864216	3	
An. gambiae	AGAP004123	425	2R	1	50350559	50354332	3	
Ae. aegypti	AAEL010626	351	supercont1.491	1	826221	794272	3	
Cu. quinquefasciatu	CPIJ006984	400	supercont3.144	1	326156	314299	3	
B. mori	BGIBMGA00001	383	nscaf1071	-1	456503	450075	4	Located after Osi24
Ap. mellifera	None							
Ca. floridanus	None							
T. castaneum	TC011655	619	ChLG9	1	20609466	20598308	6	TCOGS2:GLEAN_11655 (XP_967689 contains NPFR1+Osi24)
Ac. pisum	ACYPI007664	391	GL349633	+	115595	205777	6	
P. humanus	PHUM025830	364	DS235004.1	1	376037	377170	2	

	Osiris 24							
**Genome**	**ID**	**Length (AA)**	**Chromosome (linkage group)**	**Direct**	**Start**	**End**	**# exons**	**Note**
D. melanogaster	CG15589	533	3R	1	2030167	2032903	5	
D. pseudoobscura	GA13830	537	2	1	15796968	15795081	5	
D. virilis	GJ14246	472	scaffold_12822	1	1850145	1847962	6	
D. grimshawi	GH14017	497	scaffold_14624	1	2854383	2852256	5	
An. gambiae	AGAP004124	409	2R	1	50384354	50387218	5	
Ae. aegypti	AAEL010627	445	supercont1.491	1	449215	396633	5	
Cu. quinquefasciatu	CPIJ006981	427	supercont3.144	1	144904	128341	5	
B. mori	BGIBMGA000025	479	nscaf1071	-1	273107	262711	7	Located before NPFR1
Ap. mellifera	GB18863	473	Group15.14	1	552773	555471	4	Amel_2.0_OGSv1_gmap (XP_001121409 LG15:4223598..4226296, no longer in NCBI)
Ca. floridanus	CFLO15236	471	scaffold309	1	116909	101296	6	OGSv1.0 (EFN64650/GL441542.1)
T. castaneum	GLEAN_11656	418	ChLG9	1	20593022	20590333	4	Glean_5_19_06 (XP_967689 contains NPFR1+Osi24)
Ac. pisum	ACYPI52893	501	GL349761	1	61451	21695	7	
P. humanus	PHUM026040	295	DS235004.1	1	403565	405857	5	5'-end missing

	Osiris 2							
**Genome**	**ID**	**Length (AA)**	**Chromosome (linkage group)**	**Direct**	**Start**	**End**	**# exons**	**Note**
D. melanogaster	CG1148-PA	390	3R	1	2037184	2039100	4	isoform
	CG1148-PB	390	3R	1	2037184	2039100	4	isoform
D. pseudoobscura	GA13830	404	2	1	15789619	15788078	4	
D. virilis	GJ14247	397	scaffold_12822	1	1842804	1841325	4	
D. grimshawi	GH14018	406	scaffold_14624	1	2847740	2845905	4	
An. gambiae	AGAP004125	369	2R	1	50398489	50399671	2	
Ae. aegypti	AAEL010623	365	supercont1.491	1	232553	231401	2	missing the 3' end
Cu. quinquefasciatu	CPIJ008149	362	Supercontig 3.179	1	692137	690987	2	
B. mori	BGIBMGA000040	142	nscaf1071	1	584271	584699	1	missing 3' half, weak similarity
Ap. mellifera	GB15845	325	Group15.14	1	558576	559878	4	Amel_2.0_OGSv1_gmap (XP_001121449.1, LG15:4536711..4539563)
Ca. floridanus	CFLO15237	317	scaffold309	1	97058	94081	4	OGSv1.0 (EFN64649/GL441542.1)
T. castaneum	TC011657	319	ChLG9	1	20588465	20587053	5	TCOGS2, XP_967536.1
Ac. pisum	ACYPI007856	377	GL349761	1	7604	120	3	
P. humanus	PHUM026050	369	DS235004.1	1	413407	415968	3	

	Osiris 3							
**Genome**	**Acc#**	**Length (AA)**	**Chromosome (linkage group)**	**Direct**	**Start**	**End**	**# exons**	**Note**
D. melanogaster	CG1150	288	3R	1	2041786	2043639	3	
D. pseudoobscura	GA11035	289	2	1	15785557	15784397	3	
D. virilis	GJ14248	282	scaffold_12822	1	1837891	1836711	3	
D. grimshawi	GH14019	284	scaffold_14624	1	2842701	2841453	3	
An. gambiae	AGAP004126	295	2R	1	50423391	50425056	3	
Ae. aegypti	AAEL010624	287	supercont1.491	1	153272	137568	3	
Cu. quinquefasciatu	CPIJ008148	269	supercont3.179	1	677815	663250	3	
B. mori	BGIBMGA000042	212	nscaf1071	1	620089	626738	4	missing regions at both ends
Ap. mellifera	GB19141	294	Group15.14	1	565917	567581	2	Amel_2.0_OGSv1_gmap (XP_001121482.1 LG15:4544707..4547498)
Ca. floridanus	CFLO15238	278	scaffold309	1	85571	84078	2	OGSv1.0 (EFN64648/GL441542.1)
T. castaneum	TC011658	276	ChLG9	1	20584063	20582557	4	TCOGS2, XP_967452.1
Ac. pisum	ACYPI001904	303	GL349870	+	639943	646593	3	
P. humanus	PHUM026160	300	DS235004.1	1	431224	433412	5	

	Osiris 4							
**Genome**	**ID**	**Length (AA)**	**Chromosome (linkage group)**	**Direct**	**Start**	**End**	**# exons**	**Note**
D. melanogaster	CG10303	393	3R	-1	2047384	2044255	4	
D. pseudoobscura	GA10232	405	2	-1	15780992	15783643	4	
D. virilis	GJ14490	399	scaffold_12822	-1	1832406	1835997	4	
D. grimshawi	GH14283	403	scaffold_14624	-1	2837162	2840675	4	
An. gambiae	AGAP004127	306	2R	-1	50430646	50429581	3	
Ae. aegypti	AAEL010625	312	supercont1.491	-1	88165	101794	3	
Cu. quinquefasciatu	CPIJ008147	317	supercont3.179	-1	651118	653972	3	
B. mori	None							
Ap. mellifera	None							
Ca. floridanus	None							
T. castaneum								
Ac. pisum	None							
P. humanus	None							

	Osiris 5							
**Genome**	**ID**	**Length (AA)**	**Chromosome (linkage group)**	**Direct**	**Start**	**End**	**# exons**	**Note**
D. melanogaster	CG15590-PA	202	3R	1	2053428	2054461	2	isoform
	CG15590-PB	201	3R	1	2053428	2054458	2	isoform
D. pseudoobscura	GA13832	212	2	1	15775460	15774575	2	
D. virilis	GJ14249	210	scaffold_12822	1	1823924	1822411	2	
D. grimshawi	GH14020	219	scaffold_14624	1	2830195	2828423	2	
An. gambiae	AGAP012556	228	UNKN	-	15940121	15939378	2	
Ae. aegypti	AAEL010628	257	supercont1.491	-1	36946	50288	2	
	AAEL010631	249	supercont1.491	1	12724	1865	2	
Cu. quinquefasciatu	CPIJ008146	251	supercont3.179	1	634575	623303	2	
B. mori	BGIBMGA00004	163	nscaf1071	1	688056	693867	3	Weak similarity
Ap. mellifera	au8.g4221a	293	Group15.14	1	572124	573215	3	Based on au8.g4221.t1 ex1-ex3, (572124..572628,572715..572822,572947..573215) (XP_001121508 LG15:4551244..4552330, no
Ca. floridanus	CFLO15239	152	scaffold309	1	78191	77108	3	OGSv1.0 (EFN64647/GL441542.1) missing 5' region
T. castaneum	TC011659	229	ChLG9	1	20578022	20576613	2	5a, TCOGS2, XP_976093.1
	TC012811	232	ChLG9	-1	20581262	20582060	5	5b, TCOGS2, Osi5-like?
Ac. pisum	None							
P. humanus	None							

	Osiris 6							
Genome	ID	Length (AA)	Chromosome (linkage group)	Direct	Start	End	# exons	Note
D. melanogaster	CG1151	312	3R	1	2060458	2062024	2	
D. pseudoobscura	GA26494	288	2	1	15768074	15766825	2	
D. virilis	GJ14250	294	scaffold_12822	1	1813629	1812417	2	
D. grimshawi	GH14022	297	scaffold_14624	1	2820796	2819537	2	
An. gambiae	AGAP004129	237	2R	1	50491387	50492715	2	
Ae. aegypti	AAEL014433	240	supercont1.1116	+	181441	189732	2	
Cu. quinquefasciatus	CPIJ008144	240	supercont3.179	1	530566	525882	2	
B. mori	None							
Ap. mellifera	GB19629	257	Group15.14	1	580714	581723	3	Amel_2.0_OGSv1_gmap (XP_001121541.1 LG15:4559825..4560843)
Ca. floridanus	CFLO15240	273	scaffold309	1	70694	69570	3	OGSv1.0 (EFN64646/GL441542.1)
T. castaneum	None							
Ac. pisum	ACYPI000840	287	GL349857	-	605198	602013	4	
P. humanus	None							

	Osiris 7							
Genome	ID	Length (AA)	Chromosome (linkage group)	Direct	Start	End	# exons	Note
D. melanogaster	CG1153	288	3R	1	2074929	2075795	1	
D. pseudoobscura	GA11054	287	2	1	15754289	15753426	1	
D. virilis	GJ14251	288	scaffold_12822	1	1797298	1796432	1	
D. grimshawi	GH14023	291	scaffold_14624	1	2805530	2804655	1	
An. gambiae	AGAP004130	297	2R	1	50520138	50521031	1	
Ae. aegypti	None							
Cu. quinquefasciatus	CPIJ008141	288	supercont3.179	1	431192	430326	1	
B. mori	BGIBMGA000044	261	nscaf1071	1	709297	710082	1	
Ap. mellifera	GB13419	277	Group15.14	1	587513	589282	3	Amel_2.0_OGSv1_gmap (XP_624937.1 LG15:4566421..4568940)
Ca. floridanus	CFLO15241a	275	scaffold309	1	63969	61248	3	CFLO15241:ex1-ex3 AUGUSTUS model 61248..61630,63407..63582,63701..63969 (EFN64645=Osi7+Osi8)
T. castaneum	TC011660	278	ChLG9	1	20571810	20570974	1	TCOGS2, XP_967197.1
Ac. pisum	ACYPI002725	298	GL349857	-	577533	574085	3	
P. humanus	PHUM026170	272	DS235004.1	1	443236	446107	3	

	Osiris 8							
Genome	ID	Length (AA)	Chromosome (linkage group)	Direct	Start	End	# exons	Note
D. melanogaster	CG15591	274	3R	1	2080997	2082114	2	
D. pseudoobscura	GA13833	287	2	1	15747924	15746989	2	
D. virilis	GJ14253a	276	scaffold_12822	1	1786185	1785242	3	GJ14253+GJ14254 AUGUSTUS: 1785242..1785634,1785675..1785839,1785913..1786185
D. grimshawi	GH14024	281	scaffold_14624	1	2798067	2797160	2	
An. gambiae	AGAP004128	282	2R	1	50448638	50449565	2	extra 3 aa's before M
Ae. aegypti	AAEL004275	247	supercont1.113	-	492815	491930	2	
Cu. quinquefasciatus	CPIJ008140	280	supercont3.179	1	388401	387495	2	
B. mori	BGIBMGA000009	236	nscaf1071	-1	828580	820371	3	
Ap. mellifera	GB10057	264	Group15.14	1	592556	593593	2	Amel_2.0_OGSv1_gmap (XP_003249793.1 LG15:4571385..4573071)
Ca. floridanus	CFLO15241b	259	scaffold309	1	56944	55895	2	CFLO15241:ex4-ex5 AUGUSTUS model 55895..56178,56447..56944 (starts with 'atatt') (EFN64645=Osi7+Osi8)
T. castaneum	TC011661	254	ChLG9	1	20567569	20566676	2	TCOGS2, XP_967101.1
Ac. pisum	ACYPI005238	254	GL349857	-	550960	544609	3	
P. humanus	None							

	Osiris 9							
**Genome**	**ID**	**Length (AA)**	**Chromosome (linkage group)**	**Direct**	**Start**	**End**	**# exons**	**Note**
D. melanogaster	CG15592	233	3R	1	2086135	2087371	3	
D. pseudoobscura	GA13834	232	2	1	15742961	15741569	3	
D. virilis	GJ14255	231	scaffold_12822	1	1780683	1779314	3	
D. grimshawi	GH14025	231	scaffold_14624	1	2792525	2791171	3	
An. gambiae	AGAP004131	238	2R	1	50568998	50570643	3	
Ae. aegypti	AAEL004280	237	supercont1.113	-	282815	276138	3	
Cu. quinquefasciatus	CPIJ008139	235	supercont3.179	1	291863	289386	3	
B. moriyama	BGIBMGA000013	239	nscaf1071	-1	731188	722003	3	9f
	BGIBMGA000045	241	nscaf1071	1	756455	761654	3	9e
	BGIBMGA000012b	243	nscaf1071	-1	776934	775097	3	9d 000012 contains two Osi9 Augustus model nscaf1071:775097..775440,776272..776524,776800..776934
	BGIBMGA000012a	341	nscaf1071	-1	780388	777716	5	9c 000012 contains two Osi9 Augustus model nscaf1071:777716..778070,779043..779240,779318..779351,779496..779742,780197..780388
	BGIBMGA000011	235	nscaf1071	-1	795081	788764	3	9b
	BGIBMGA000010	240	nscaf1071	-1	813664	807853	3	9a
Ap. mellifera	GB19626	255	Group15.14	1	599571	600600	3	Amel_2.0_OGSv1_gmap (XP_001121625.1 LG15:4578466..4580519)
Ca. floridanus	CFLO15242	250	scaffold309	1	49954	48426	3	OGSv1.0 (EFN64644/GL441542.1)
T. castaneum	TC011662	250	ChLG9	1	20564637	20562939	3	TCOGS2, XP_001808361.1
Ac. pisum	ACYPI004638	264	GL349857	-	502336	498348	2	
P. humanus	PHUM026280	258	DS235004.1	1	457230	458434	3	

	Osiris 10							
Genome	ID	Length (AA)	Chromosome (linkage group)	Direct	Start	End	# exons	Note
D. melanogaster	CG15593-PB	741	3R	1	2089581	2093117	4	possibly 10a+10b
	CG15593-PA	576	3R	1	2089698	2093117	5	possibly 10a+10b
D. pseudoobscura	GA13835-PC	634	2	1	15738413	15735593	5	possibly 10a+10b
	GA13835-PB	767	2	1	15738488	15735593	4	possibly 10a+10b
D. virilis	GJ14257	592	scaffold_12822	1	1775378	1771016	5	possibly 10a+10b
D. grimshawi	GH14026	509	scaffold_14624	1	2787800	2784545	5	possibly 10a+10b missing 5' end
An. gambiae	AGAP004132	610	2R	1	50579082	50585938	5	possibly 10a+10b
Ae. aegypti	AAEL004303	565	supercont1.113	-	205352	160117	5	possibly 10a+10b
Cu. quinquefasciatus	CPIJ008138	564	supercont3.179	1	260063	230396	5	possibly 10a+10b
B. mori	BGIBMGA000046	314	nscaf1071	1	839498	846172	4	10a or 10b only
Ap. mellifera	XP_003249790.1	314	Group15.14	1	602439	603849	2	10a, NCBI_RefSeq
	GB13856	223	Group15.14	1	606624	607462	3	10b, Amel_2.0_OGSv1_gmap
Ca. floridanus	CFL015243	283	scaffold309	1	46630	44997	2	10a, OGSv1.0 (EFN64643/GL441542.1)
	CFLO15244	219	scaffold309	1	40662	39172	3	10b, OGSv1.0 (EFN64644/GL441542.1)
T. castaneum	XP_967021.2	533	ChLG9	1	20561128	20557379	8	LOC655388, TC011663+TC011664 possibly 10a+10b
Ac. pisum	ACYPI37205	360	GL349857	-	494658	484212	4	10a
	ACYPI37204	240	GL349857	+	477903	479043	2	10b
P. humanus	PHUM026300	307	DS235004.1	1	470753	471897	4	10a
	PHUM026310	165	DS235004.1	1	476,424	477503	4	10b

	Osiris 11							
Genome	ID	Length (AA)	Chromosome (linkage group)	Direct	Start	End	# exons	Note
D. melanogaster	CG15596	302	3R	-1	2094410	2093502	1	
D. pseudoobscura	GA13838	317	2	-1	15734475	15735428	1	
D. virilis	GJ14489	321	scaffold_12822	-1	1769902	1770867	1	
D. grimshawi	GH14282	326	scaffold_14624	-1	2783391	2784371	1	
An. gambiae	AGAP004134	263	2R	1	50594209	50595000	1	
Ae. aegypti	AAEL004298	263	supercont1.113	+	85610	86401	1	
Cu. quinquefasciatu	CPIJ008136	262	supercont3.179	1	198762	197867	1	
B. mori	BGIBMGA00000	253	nscaf1071	-1	851744	850983	1	
Ap. mellifera	XP_003249794.	202	Group15.14	-1	609315	608097	3	NCBI-RefSeq model corresponding to GB14757, but without extra 12 aa at 5' end
Ca. floridanus	CFLO15250	193	scaffold309	-1	35342	37142	3	OGSv1.0, missing 5' region (EFN64641/GL441542.1)
T. castaneum	TC012810	279	ChLG9	-1	20555016	20556390	2	TCOGS2 (XP_996928.2 missing 5' and 3')
Ac. pisum	ACYPI003143	316	GL349857	-	465923	448880	5	
P. humanus	PHUM026320	346	DS235004.1	-1	482961	481380	3	

	Osiris 12							
**Genome**	**ID**	**Length (AA)**	**Chromosome (linkage group)**	**Direct**	**Start**	**End**	**# exons**	**Note**
D. melanogaster	CG1154	295	3R	1	2104429	2107526	2	
D. pseudoobscura	GA11059	310	2	1	15724932	15722318	2	
D. virilis	GJ14258	296	scaffold_12822	1	1759247	1755541	2	
D. grimshawi	GH14027	306	scaffold_14624	1	2773783	2770028	2	
An. gambiae	AGAP013195	245	2R	1	50611644	50612588	2	
Ae. aegypti	AAEL002957a	232	supercont1.73	+	533897	545601	2	based on AAEL002957 ex4-ex5:
Cu. quinquefasciatu	CPIJ008135	342	supercont3.179	1	128851	120079	5	
B. mori	BGIBMGA000047	234	nscaf1071	1	860527	877819	3	BGIBMGA00004 missing 3' end; Augustus model nscaf1071:860527..860929,866403..866530,877646..877819
Ap. mellifera	XP_001121769.1	263	Group15.14	1	614359	615150	1	NCBI_RefSeq model corresponding to GB18845, but with extra 41 aa at N-term (LG15:4593479..4594270)
Ca. floridanus	CFLO15245	257	scaffold309	1	31139	30366	1	OGSv1.0 (EFN64640/GL441542.1)
T. castaneum	TC011665	237	ChLG9	1	20552342	20551573	2	TCOGS2, XP_966834.1
Ac. pisum	ACYPI000603	255	GL349857	+	445712	449651	2	
P. humanus	None							

	Osiris 13							
Genome	ID	Length (AA)	Chromosome (linkage group)	Direct	Start	End	# exons	Note
D. melanogaster	CG15595	210	3R	1	2117624	2118491	2	
D. pseudoobscura	GA13837	219	2	1	15713662	15712936	2	
D. virilis	GJ14260	207	scaffold_12822	1	1745092	1744398	2	
D. grimshawi	GH14029	208	scaffold_14624	1	2759717	2758601	2	
An. gambiae	None							
Ae. aegypti	None							
Cu. quinquefasciatu	None							
B. mori	BGIBMGA00004	222	nscaf1071	1	889090	891513	3	Weak similarity
Ap. mellifera								
Ca. floridanus	CFLO15246	225	scaffold309	1	23657	21613	3	OGSv1.0 (EFN64638/GL441542.1) weak similarity
T. castaneum	None							
Ac. pisum	None							
P. humanus	None							

	Osiris 14							
Genome	ID	Length (AA)	Chromosome (linkage group)	Direct	Start	End	# exons	Note
D. melanogaster	CG1155	268	3R	1	2124973	2126299	3	
D. pseudoobscura	GA11061	269	2	1	15707310	15706315	3	
D. virilis	GJ14261	277	scaffold_12822	1	1737239	1736231	3	
D. grimshawi	GH14030	270	scaffold_14624	1	2750767	2749290	3	
An. gambiae	AGAP003465	280	2R	1	38016606	38018316	3	
Ae. aegypti	AAEL002962	290	supercont1.73	+	921829	928099	3	
Cu. quinquefasciatu	CPIJ009837	273	supercont3.253	-	539625	536876	3	
B. mori	BGIBMGA00005	189	nscaf1071	1	899025	905630	4	
Ap. mellifera	GB19255	267	Group15.14	1	625746	627315	3	Amel_2.0_OGSv1_gmap (XP_392084.1 LG15:4604768..4606632)
Ca. floridanus	CFLO15247	262	scaffold309	1	18470	16528	3	OGSv1.0 (EFN64637/GL441542.1)
T. castaneum	TC011827	245	ChLG9	1	17784120	17783271	3	TCOGS2, XP_972144.1
Ac. pisum	ACYPI010031	258	GL349857	-	367021	365776	3	
P. humanus	PHUM026630	271	DS235004.1	1	514933	515929	3	

	Osiris 15							
**Genome**	**ID**	**Length (AA)**	**Chromosome (linkage group)**	**Direct**	**Start**	**End**	**# exons**	**Note**
D. melanogaster	CG1157	214	3R	1	2127635	2128897	3	
D. pseudoobscura	GA11070	214	2	1	15704652	15703720	3	
D. virilis	GJ14262	215	scaffold_12822	1	1734711	1733651	3	
D. grimshawi	GH14031	214	scaffold_14624	1	2747671	2746647	3	
An. gambiae	AGAP003466	206	2R	1	38023425	38027635	3	
Ae. aegypti	AAEL002949	206	supercont1.73	+	947347	977880	3	
Cu. quinquefasciatu	CPIJ009835	208	supercont3.253	-	520208	503607	3	
B. mori	None							
Ap. mellifera	GB14511	233	Group15.14	1	629349	631777	2	Amel_2.0_OGSv1_gmap (XP_001121861.1 LG15:4608426..4611097)
Ca. floridanus	CFLO15248	234	scaffold309	1	14885	12348	2	OGSv1.0 (EFN64636/GL441542.1)
T. castaneum	TC011828	188	ChLG9	1	17771168	17770175	2	TCOGS2, XP_971987.1
Ac. pisum	None							
P. humanus	None							

	Osiris 16							
**Genome**	**ID**	**Length (AA)**	**Chromosome (linkage group)**	**Direct**	**Start**	**End**	**# exons**	**Note**
D. melanogaster	CG31561	278	3R	1	2130992	2131887	2	
D. pseudoobscura	GA16326	277	2	1	15701265	15700368	2	
D. virilis	GJ14264	278	scaffold_12822	1	1731967	1730888	2	
D. grimshawi	GH14033	276	scaffold_14624	1	2745403	2744257	2	
An. gambiae	AGAP003467	238	2R	1	38030429	38031217	2	16a
	AGAP003472	238	2R	1	38077169	38077957	2	16b
Ae. aegypti	AAEL002961	254	supercont1.73	+	1025518	1038012	2	
Cu. quinquefasciatu	CPIJ009834	243	supercont3.253	-	495771	494975	2	
B. mori	BGIBMGA00005	248	nscaf1071	1	916070	920540	2	16a
	BGIBMGA00005	222	nscaf1071	1	924686	927766	2	16b
	See Osiris-like located here							
Ap. mellifera	GB16524	303	Group15.14	1	636935	638007	2	Amel_2.0_OGSv1_gmap (XP_001121887.1 includes extra 3')
Ca. floridanus	CFLO15249	294	scaffold309	1	6971	5865	2	OGSv1.0 (EFN64635/GL441542.1)
T. castaneum	TC012679	257	ChLG9	-1	17774547	17775320	1	16b, TCOGS2, XP_972042.1
	TC012680	260	ChLG9	-1	17778206	17781508	2	16a, TCOGS2, XP_972093.1
Ac. pisum	ACYPI37215	395	GL349857	-	264785	258197	3	16c
	ACYPI37213	335	GL349857	-	335265	325498	3	16b
	ACYPI37212	206	GL349857	-	347582	342664	3	16a
	ACYPI37197	271	GL349857	+	386913	389207	2	16f, located before Osi14
	ACYPI37210	284	GL349857	-	398236	395754	2	16e, located before Osi14
	ACYPI007501	287	GL349857	-	402294	399369	2	16d, located before Osi14
P. humanus	None							

	Osiris 17							
**Genome**	**ID**	**Length (AA)**	**Chromosome (linkage group)**	**Direct**	**Start**	**End**	**# exons**	**Note**
D. melanogaster	CG15598	648	3R	1	2141609	2152519	8	
D. pseudoobscura	GA26496-PA	740	2	1	15692057	15683149	9	Isoforms
	GA26496-PC	654	2	1	15692183	15683149	9	Isoforms
D. virilis	GJ14266	705	scaffold_12822	1	1717024	1703545	9	
D. grimshawi	GH14034a	761	scaffold_14624	1	2735733	2723660	9	last exon removed. Revised model: (2723660..2723801,2726507..2726685,2726749..2726859,2728780..2728978,2729765..2730073,2730663..2730850,2733027..2733280,2733378..2733611,2735064..2735
An. gambiae	AGAP003468	661	2R	1	38042427	38056441	8	
Ae. aegypti	AAEL002966a	598	supercont1.73	+	1118311	1246764	8	AAEL002966 + AAEL002952 genewise model: CDS(1118311..1118617, 1155934..1156176,1166316..1166569,1182188..1182342,1183777..1184010, 1193705..1193876, 1215056..1215336, 1246614..1246764), start codon not found
Cu. quinquefasciatu	CPIJ009830a	669	supercont3.253	-	449432	366128	8	CPIJ009830+CPIJ009831+CPIJ009832+CPIJ009833; genewise model: CDS(449432..449153,417630..417388, 413069..412816,406802..406639,403596..403396,393345..393201, 379175..378895, 366278..366128); Need to check
B. mori	BGIBMGA000054	588	nscaf1071	1	968425	985551	10	
Ap. mellifera	XP_001121915.2	586	Group15.14	1	655434	658194	4	NCBI-RefSeq model corresponding to GB16817, but with extra 91 aa at N-term (LG15:4634123..4638274)
Ca. floridanus	CFLO14498	560	scaffold767	-	348549	345974	5	OGSv1.0 (EFN71496/GL436778.1)
T. castaneum	None							
Ac. pisum	ACYPI006202	525	GL349975	+	190119	206662	7	
P. humanus	PHUM497620a	645	DS235830.1	-	141828	133937	8	PHUM497620+PHUM497610; GeneWise model: 141828..141297, 138866..138492, 135455..135226, 135153..134991, 134907..134726, 134649..134541, 134461..134257, 134078..133937

	Osiris 18							
**Genome**	**ID**	**Length (AA)**	**Chromosome (linkage group)**	**Direct**	**Start**	**End**	**# exons**	**Note**
D. melanogaster	CG1169	306	3R	1	2156073	2157285	2	
D. pseudoobscura	GA11143	313	2	1	15676247	15675240	2	
D. virilis	GJ14267	309	scaffold_12822	1	1700037	1699044	2	
D. grimshawi	GH14035	318	scaffold_14624	1	2720195	2719171	2	
An. gambiae	AGAP003469	265	2R	-1	38058328	38057430	2	
	AGAP012548	106	UNKN	-1	14659597	14654022	2	Almost identical copy in unknown location; possible assembly mistake; see
Ae. aegypti	AAEL002965	266	supercont1.73	-	1259787	1258929	2	
Cu. quinquefasciatu	CPIJ009829	267	supercont3.253	+	356854	357720	2	
B. mori	BGIBMGA00000	270	nscaf1071	-1	993294	992334	3	
Ap. mellifera	GB16900	249	Group15.14	1	659884	660816	3	Amel_2.0_OGSv1_gmap (XP_001121942.2 LG15:4638990..4640092)
Ca. floridanus	CFLO14499	246	scaffold767	-	342467	341404	3	OGSv1.0 (EFN71495/GL436778.1)
T. castaneum	TC012820	246	ChLG9	-1	20684703	20685544	3	TCOGS2, XP_968992.1
Ac. pisum	ACYPI009026	242	GL349975	+	217232	219193	4	
P. humanus	PHUM497600	285	DS235830.1	+	123337	124403	3	

	Osiris 19							
Genome	ID	Length (AA)	Chromosome (linkage group)	Direct	Start	End	# exons	Note
D. melanogaster	CG15189-PA	266	3R	1	2161165	2162662	4	isoform
	CG15189-PB	257	3R	1	2161165	2162659	4	isoform
D. pseudoobscura	GA13557	273	2	1	15672361	15670940	4	
D. virilis	GJ14268	266	scaffold_12822	1	1695141	1693812	4	
D. grimshawi	GH14036	262	scaffold_14624	1	2716195	2714924	4	
An. gambiae	AGAP003470	232	2R	1	38061429	38062320	3	
	AGAP012549	247	UNKN	+	14662691	14663580	3	Almost identical copy in unknown location; possible assembly mistake; see
Ae. aegypti	AAEL002960	250	supercont1.73	+	1285435	1286307	3	
Cu. quinquefasciatu	CPIJ009828	251	supercont3.253	-	344600	343725	3	
B. mori	BGIBMGA01071	258	nscaf3003	-	3992132	3991021	3	
Ap. mellifera	GB16804	247	Group15.14	1	663088	663932	2	Amel_2.0_OGSv1_gmap (XP_001121961.1 LG15:4642085..4643841)
Ca. floridanus	CFLO14500	247	scaffold767	-	339201	338256	2	OGSv1.0 (EFN71494/GL436778.1)
T. castaneum	TC012821	243	ChLG9	-1	20686916	20687749	3	19a, TCOGS2, XP_969068.1
	TC012822	196	ChLG9	-1	20689368	20690009	2	19b, TCOGS2, XP_969215.1
Ac. pisum	ACYPI000796	190	GL349975	+	226304	227943	4	
P. humanus	PHUM497590	255	DS235830.1	-	122029	121084	3	

	Osiris 20							
**Genome**	**ID**	**Length (AA)**	**Chromosome (linkage group)**	**Direct**	**Start**	**End**	**# exons**	**Note**
D. melanogaster	CG15188	280	3R	1	2165910	2166818	2	
D. pseudoobscura	GA13556	276	2	1	15666849	15665952	2	
D. virilis	GJ14269	277	scaffold_12822	1	1689890	1688986	2	
D. grimshawi	GH14037	280	scaffold_14624	1	2711690	2710778	2	
An. gambiae	AGAP003471	320	2R	1	38065791	38066897	2	
Ae. aegypti	AAEL002955	292	supercont1.73	+	1305642	1313330	2	
	AAEL014727	292	supercont1.1240	-	188349	182326	2	20b, Unlinked Osi20 copy; almost identical
Cu. quinquefasciatu	CPIJ009827	297	supercont3.253	-	330657	327540	2	
B. mori	BGIBMGA010717	293	nscaf3003	-	3982740	3981708	3	
Ap. mellifera	XP_001121985.1	270	Group15.14	1	665962	666847	2	NCBI_RefSeq model corresponding to GB15865, but with extra 120 aa at 5' end (LG15:4644922..4646726)
Ca. floridanus	CFLO14501	267	scaffold767	-	335705	334509	2	OGSv1.0 (EFN71493/GL436778.1)
T. castaneum	TC012823	248	ChLG9	-1	20691474	20692314	3	TCOGS2, XP_969284.1
Ac. pisum	ACYPI007146	281	GL349975	+	238670	239799	2	
P. humanus	PHUM497570	290	DS235830.1	-	117238	116133	4	

	Osiris 22							
Genome	ID	Length (AA)	Chromosome (linkage group)	Direct	Start	End	# exons	Note
D. melanogaster	CG8644	332	3R	1	9116438	9117497	2	
D. pseudoobscura	GA21234	342	2	1	9987757	9986666	2	
D. virilis	GJ10414	363	scaffold_12855	-	3036310	3035156	2	
D. grimshawi	GH18427	339	scaffold_14906	-	5747016	5745936	2	
An. gambiae	AGAP003420	366	2R	1	37510453	37511640	2	
Ae. aegypti	AAEL007198	359	supercont1.243	+	914044	915223	2	
Cu. quinquefasciatus	CPIJ011100	345	supercont3.316	+	372903	374003	2	
B. mori	BGIBMGA010678	163	nscaf2998	+	1317487	1322074	2	Middle region missing
Ap. mellifera	None							
Ca. floridanus	None							
T. castaneum	TC002385	151	unknown	+	15336400	15336898	2	Middle region missing
Ac. pisum	ACYPI002813	196	GL350227	-	167081	164772	3	Middle region
P. humanus	None							

	Osiris 23							
Genome	ID	Length (AA)	Chromosome (linkage group)	Direct	Start	End	# exons	Note
D. melanogaster	CG15538	250	3R	-1	26270756	26269871	3	
D. pseudoobscura	GA13796	270	2	-1	1254748	1255676	3	
D. virilis	GJ22762	265	scaffold_13047	-	15824251	15823319	3	
D. grimshawi	GH17432	264	scaffold_14830	+	4185984	4186905	3	
An. gambiae	AGAP000957	293	X	-	18368810	18367147	4	
Ae. aegypti	None							Short similariy against AGAP000957 and CG15538 found in supercont1.202:1394474 1394301
Cu. quinquefasciatus	Cqui_Osi23	189	supercont3.42	+	474599	480673	3	GeneWise+Augustus model: 474599..474855,474955..475082, 480489..480673; still missing 3'
B. mori	None							
Ap. mellifera	XP_001120227.2	254	Group8.6	-	87657	86288	3	NCBI_RefSeq model corresponding to GB14285, but with extra 14 aa at N-term (LG8:2309823..2307814)
Ca. floridanus	CFLO22710	249	scaffold799		210020	211808	3	OGSv1.0 (EFN70025/GL437711.1)
T. castaneum	TC030703a	226	ChLG2	+	11496092	11497260	3	TC030703:TCOGS2 with extra N/C-term. XP_970499.1 (non-Osi protein) includes this as part of its exons. AUGUSTUS model: 11496092..11496258,11496701..11496798,11496845..11497260
Ac. pisum	ACYPI50421	217	GL350440	-	80703	67062	3	
P. humanus	None							

	Osiris 21							
**Genome**	**ID**	**Length (AA)**	**Chromosome (linkage group)**	**Strand**	**Start**	**End**	**# exons**	**Note**
D. melanogaster	CG14925	282	2L	-	11285603	11284586	2	
D. pseudoobscura	GA13356	280	4_group3	-	6710686	6709780	2	
D. virilis	GJ14913	277	scaffold_12963	+	2228486	2229378	2	
D. grimshawi	GH13529	291	scaffold_15126	+	1313369	1314317	2	
An. gambiae	AGAP005899	256	2L	-	23383765	23382399	3	21a
	AGAP010354	242	3L	-	2060900	2060107	2	21b
Ae. aegypti	AAEL004788	264	supercont1.130	+	1236881	1240903	3	21a
	AAEL014873	242	supercont1.1318	-	61163	25952	2	21b
Cu. quinquefasciatus	CPIJ006501	255	supercont3.125	-	457478	456268	2	21c
	CPIJ016349	255	supercont3.798	+	131376	132525	2	21b
	CPIJ004911	270	supercont3.80	+	580797	581778	3	21a
B. mori	BGIBMGA013568a	216	nscaf3078	+	404693	405837	4	BGIBMGA013568+BGIBMGA013569 GeneWise model: 404693..404875,404959..405151, 405257..405437,405754..405837; need to check
Ap. mellifera	None							
Ca. floridanus	None							
T. castaneum	TC013496	250	ChLG5	-	10767500	10766748	1	TCOGS2, XP_976416.1
Ac. pisum	ACYPI008994	252	GL350028	-	167549	164571	2	21c
	ACYPI43145	245	GL350028	-	154956	151423	2	21b
	ACYPI005783	258	GL350028	-	135395	126447	2	21a
	ACYPI064603	141	GL356106	+	0	3876	2	21a2, missing 5' and 3'-ends
P. humanus	None							

	Osiris like							
Genome	ID	Length (AA)	Chromosome (linkage group)	Direct	Start	End	# exons	Note
D. melanogaster								
D. pseudoobscura								
D. virilis								
D. grimshawi								
An. gambiae								
Ae. aegypti								
Cu. quinquefasciatus								
B. mori	BGIBMGA000053	493	nscaf1071	1	931016	939783	7	located after Osi16b
Ap. mellifera								
Ca. floridanus								
T. castaneum								
Ac. pisum	ACYPI000560	419	GL349857	+	296605	308820	5	Located between 16b and c
	ACYPI37193	286	GL349857	+	266124	275397	3	Located between 16b and c
	ACYPI33621	262	GL350199	+	284013	299232	2	
P. humanus	PHUM025350	285	DS235004.1	-1	94973	93537	3	
	PHUM025360	360	DS235004.1	-1	110230	108271	4	
	PHUM465410	327	DS235812.1	-1	294251	290973	3	

	CG15594 like							
**Genome**	**ID**	**Lengt h (AA)**	**Chromosome (linkage group)**	**Direct**	**Start**	**End**	**# exons**	**Note**
D. melanogaster								
D. pseudoobscura								
D. virilis								
D. grimshawi								
An. gambiae								
Ae. aegypti								
Cu. quinquefasciatus								
B. mori	BGIBMGA000048272		nscaf1071	1	883002	886205	2	
Ap. mellifera	XP_003249795.1	169	Group15.14	-1	617796	616879	2	LG15:4597217..459575
Ca. floridanus	CFLO10930	225	scaffold309	-1	27850	28890	2	EFN64639/GL441542
T. castaneum								
Ac. pisum	ACYPI007103	364	GL349679	-1	542850	539522	6	
P. humanus								
