# Supplementary material for: Evolution of a Large, Conserved, and Syntenic Gene Family in Insects
Source: G3 (Bethesda). 2012 Feb 1;2(2):313–9. doi: 10.1534/g3.111.001412 (PMC3284338; doi:10.1534/g3.111.001412)
Supplement: Supporting Information [file supp_2.2.313_TableS3.pdf]

**Table S3 The list of 24 *Osiris* genes in the *D. melanogaster* genome and those identified from the 11 other *Drosophila* genomes.**

| | *D. melanogaster* | *D. simulans* | *D. sechellia* | *D. yakuba* | *D. erecta* | *D. ananassae* |
|---|---|---|---|---|---|---|
| *Osi1* | CG15585 | GD19841 | GM10858 | GE10183 | GG13122 | GF16371 |
| *Osi2* | CG1148 | GD19845 | GM10863 | GE10186 | GG13136 | GF16375 |
| *Osi3* | CG1150 | GD19846 | GM10864 | GE10187 | GG13139 | GF16376 |
| *Osi4* | CG10303 | GD19554 | GM10564 | GE24111 | GG10555 | GF18625 |
| *Osi5* | CG15590 | GD19847 | GM10866 | GE10188 | GG13143 | GF16377 |
| *Osi6* | CG1151 | GD19848 | GM10867 | GE10189 | GG13147 | GF16378 |
| *Osi7* | CG1153 | Dsim 7[‡] | GM10868 | GE10190 | GG13151 | GF16379 |
| *Osi8* | CG15591 | GD19849 | GM10869 | GE10191 | GG13155 | GF16380 |
| *Osi9* | CG15592 | GD19850 | GM10870 | GE10192 | GG13161 | GF16381 |
| *Osi10* | CG15593* | GD19851 | GM10871 | GE10194 | GG13170 | GF16382 |
| *Osi11* | CG15596 | GD19553 | GM10562 | GE24110 | GG10544 | GF18624 |
| *Osi12* | CG1154 | GD19852 | GM10872 | GE10195 | GG13181 | GF16383 |
| *Osi13* | CG15595 | GD19856 | GM10874 | GE10197 | GG13203 | GF16385 |
| *Osi14* | CG1155 | GD19857 | GM10875 | GE10198 | GG13215 | GF16386 |
| *Osi15* | CG1157 | GD19858 | GM10877 | GE10199 | GG13226 | GF16387 |
| *Osi16* | CG31561 | GD19859 | GM10879 | GE10201 | GG13248 | GF16388 |
| *Osi17* | CG15598 | GD19854 | GM10881 | GE10205 | GG13280 | GF16390 |
| *Osi18* | CG1169 | GD19861 | GM10882 | GE10206 | GG13291 | GF16391 |
| *Osi19* | CG15189 | GD19862 | GM10883 | GE10207 | GG13302 | GF16392 |
| *Osi20* | CG15188 | GD19863 | GM10884 | GE10208 | GG13313 | GF16393 |
| *Osi21* | CG14925 | GD22180 | GM11091 | GE12926 | GG10314 | GF15124 |
| *Osi22* | CG8644 | GD18894 | GM24095 | GE26258 | GG19544 | GF17589 |
| *Osi23* | CG15538 | GD17079 | GM12155 | GE23387 | GG11936 | GF23337 |
| *Osi24* | CG15589 | GD19844 | GM10862 | GE10185 | GG13130 | GF16374 |

| | D. persimilis | D. pseudoobscura | D. willistoni | D. virilis | D. mojavensis | D. grimshawi |
|---|---|---|---|---|---|---|
| *Osi1* | GL24048 | GA13829 | GK13029 | GJ14243 | GI24386 | GH14014**† |
| *Osi2* | GL24055 | GA11025 | GK13033 | GJ14247 | GI24391 | GH14018 |
| *Osi3* | GL24056 | GA11035 | GK13034 | GJ14248 | GI24392 | GH14019 |
| *Osi4* | GL23480 | GA10232 | GK14207 | GJ14490 | GI23175 | GH14283 |
| *Osi5* | GL24057 | GA13832 | GK13035 | GJ14249 | GI24393 | GH14020 |
| *Osi6* | GL24058 | GA26494 | GK13036 | GJ14250 | GI24394 | GH14022 |
| *Osi7* | GL24059 | GA11054 | GK13037 | GJ14251 | GI24395 | GH14023 |
| *Osi8* | GL24060 | GA13833 | GK13038 | GJ14253** | GI24396 | GH14024 |
| *Osi9* | GL24061 | GA13834 | Dwil 9# | GJ14255 | GI24397 | GH14025 |
| *Osi10* | GL24062 | GA13835 | GK13040 | GJ14257 | GI24398 | GH14026 |
| *Osi11* | GL23479 | GA13838 | GK14206 | GJ14489 | GI23173 | GH14282 |
| *Osi12* | GL24063 | GA11059 | GK13041 | GJ14258 | GI24400 | GH14027 |
| *Osi13* | GL24066 | GA13837 | GK13043 | GJ14260 | GI24402 | GH14029 |
| *Osi14* | GL24067 | GA11061 | GK13044 | GJ14261 | GI24403 | GH14030 |
| *Osi15* | GL24068 | GA11070 | GK13045 | GJ14262 | GI24404 | GH14031 |
| *Osi16* | GL24070 | GA16326 | GK13048 | GJ14264 | GI24405 | GH14033 |
| *Osi17* | GL24071 | GA26496 | GK13049 | GJ14266 | GI24407 | GH14034** |
| *Osi18* | GL24072 | GA11143 | GK13051 | GJ14267 | GI24408 | GH14035 |
| *Osi19* | GL24073 | GA13557 | GK13052 | GJ14268 | GI24409 | GH14036 |
| *Osi20* | GL24074 | GA13556 | GK13053 | GJ14269 | GI24411 | GH14037 |
| *Osi21* | GL18575 | GA13356 | GK21104 | GJ14913 | GI18143 | GH13529 |
| *Osi22* | GL24463 | GA21234 | GK13933 | GJ10414 | GI24328 | GH18427 |
| *Osi23* | GL14048 | GA13796 | GK13129 | GJ22762 | GI22761 | GH17432 |
| *Osi24* | GL24053 | GA13830 | GK13032 | GJ14246 | GI24390 | GH14017 |

**Osi10* has two alternative transcripts. For our alignment and phylogenetic analysis, Osi10-PA (CG15593-PA) protein sequence was used.

**The annotations for these genes are corrected in this study. See Supplementary Table S2 for details.

†These genes are not annotated as *Osiris* orthologs in Flybase.

‡*D. simulans Osi7* is not annotated in FlyBase, but the gene is identified in the genome (3R:2090001..2094500).

#*D. willistoni Osi9* is not annotated in FlyBase, but the gene is identified in the initial region of GK13040.
