# Supplementary material for: Evolution of a Large, Conserved, and Syntenic Gene Family in Insects
Source: G3 (Bethesda). 2012 Feb 1;2(2):313–9. doi: 10.1534/g3.111.001412 (PMC3284338; doi:10.1534/g3.111.001412)
Supplement: Supporting Information [file supp_2.2.313_TableS4.pdf]

Table S4 The list of data sources **for the arthropod genomes used in this study.**

Species	Source	Release
Drosophila melanogaster	FlyBase (http://flybase.org/)	R5.40
Drosophila simulans	FlyBase (http://flybase.org/)	R1.3
Drosophila sechellia	FlyBase (http://flybase.org/)	R1.3
Drosophila yakuba	FlyBase (http://flybase.org/)	R1.3
Drosophila erecta	FlyBase (http://flybase.org/)	R1.3
Drosophila ananassae	FlyBase (http://flybase.org/)	R1.3
Drosophila pseudoobscura	FlyBase (http://flybase.org/)	R2.23
Drosophila persimilis	FlyBase (http://flybase.org/)	R1.3
Drosophila willistoni	FlyBase (http://flybase.org/)	R1.3
Drosophila mojavensis	FlyBase (http://flybase.org/)	R1.3
Drosophila virilis	FlyBase (http://flybase.org/)	R1.3
Drosophila grimshawi	FlyBase (http://flybase.org/)	R1.3
Anopheles gambiae	VectorBase (http://www.vectorbase.org/SequenceData/)	AgamP3.6
Aedes aegypti	VectorBase (http://www.vectorbase.org/SequenceData/)	AaegL1.2
Culex quinquefasciatus	VectorBase (http://www.vectorbase.org/SequenceData/)	CpipJ1.2
Bombyx mori	Silkworm Genome Database (http://silkworm.genomics.org.cn)	

		v2.0
Apis mellifera	BeeBase (http://hymenopteragenome.org/beebase/)	Amel_4.5
Camponotus floridanus	Ant Genomes Portal (http://hymenopteragenome.org/ant_genomes/)	V3.3
Tribolium castaneum	BeetleBase (http://beetlebase.org/)	Tcas_3.0
Acyrthosiphon pisum	AphidBase (http://www.aphidbase.com/aphidbase)	Acyr_2.0
Pediculus humanus	VectorBase (http://www.vectorbase.org/SequenceData/)	PhumU1.2
Daphnia pulex	Joint Genome Institute (http://www.jgi.doe.gov)	v1.0
Ixodes scapularis	VectorBase (http://www.vectorbase.org/SequenceData/)	IscaW1.1
